# Growth temperature and chromatinization in archaea

**DOI:** 10.1038/s41564-022-01245-2

**Published:** 2022-10-20

**Authors:** Antoine Hocher, Guillaume Borrel, Khaled Fadhlaoui, Jean-François Brugère, Simonetta Gribaldo, Tobias Warnecke

**Affiliations:** 1 Medical Research Council London Institute of Medical Sciences, London, UK; 2 Institute of Clinical Sciences, Faculty of Medicine, Imperial College London, London, UK; 3 Institut Pasteur, Université Paris Cité, UMR CNRS6047, Evolutionary Biology of the Microbial Cell, Paris, France; 4 Université Clermont Auvergne, CNRS, Lab Microorganismes: Génome et Environnement LMGE, Clermont-Ferrand, France

**Keywords:** Microbiology, Molecular evolution

## Abstract

DNA in cells is associated with proteins that constrain its structure and affect DNA-templated processes including transcription and replication. HU and histones are the main constituents of chromatin in bacteria and eukaryotes, respectively, with few exceptions. Archaea, in contrast, have diverse repertoires of nucleoid-associated proteins (NAPs). To analyse the evolutionary and ecological drivers of this diversity, we combined a phylogenomic survey of known and predicted NAPs with quantitative proteomic data. We identify the Diaforarchaea as a hotbed of NAP gain and loss, and experimentally validate candidate NAPs in two members of this clade, *Thermoplasma volcanium* and *Methanomassiliicoccus luminyensis*. Proteomic analysis across a diverse sample of 19 archaea revealed that NAP investment varies from <0.03% to >5% of total protein. This variation is predicted by growth temperature. We propose that high levels of chromatinization have evolved as a mechanism to prevent uncontrolled helix denaturation at higher temperatures, with implications for the origin of chromatin in both archaea and eukaryotes.

## Abstract (web summary)

A systematic phylogenomic survey finds that growth temperature is a good quantitative predictor of nucleoid-associated protein abundance.

## Main

Archaeal genomes contain small, abundant, often basic, proteins that bind DNA with low sequence specificity and are known as nucleoid-associated proteins (NAPs). Several proteins that fit this description have been reported in archaeal model organisms, and include Alba, Cren7, MC1 and histones^1^. Whereas histones provide the backbone of chromatin across eukaryotes, the repertoire of major chromatin proteins in archaea is considerably more diverse. Histones are absent from several lineages, including the Sulfolobales/Desulfurococcales and Parvarchaeota^2^. Several NAPs are abundant but lineage specific, including HTa in the Thermoplasmatales^3^ and Sul7 in the Sulfolobales^1^.

The evolutionary and ecological drivers of NAP gain and loss in archaea are poorly understood^4^. Do different NAPs represent adaptations to specific niches? If so, what factors determine the presence or absence of a given NAP in a given genome? Alternatively, are NAPs in archaea diverse because several different proteins can do the same job, rendering them interchangeable?

Phylogenomics charts the distribution of homologous genes across a set of genomes and enables gain and loss events to be traced along a phylogeny. The resulting presence/absence patterns, integrated with ecological contexts, may reveal clues as to why a particular protein is found in one set of genomes but not another. Phyletic comparisons, however, can be treacherous. The presence of a specific gene in two genomes does not necessarily imply that the protein product is doing the same job in both. Histones, for example, are highly abundant at the protein level in *Thermococcus kodakarensis* (1.76% of total protein; see below for how relative abundance is calculated) but only weakly expressed in *Halobacterium salinarum* (0.02% of total protein)^5–7^. Given this difference in abundance, histones are unlikely to have the same roles in nucleoid biology in these two species. Consistent with this, retention of at least one of its two histone genes (*htkA* and *htkB*) is essential in *T. kodakarensis*^8^ whereas the single *H. salinarum* histone gene (*hpyA*) is dispensable for growth^7^ and binds to fewer than 60 sites along the chromosome^9^. Alba, too, is highly expressed in many archaea, including *Sulfolobus shibatae* (1.6% of total protein) but >100-fold less abundant in others, for example, *Methanococcus maripaludis* (0.01% of total protein)^10^. These large differences in abundance are indicative of cryptic functional diversity that is not directly accessible via comparative genomics.

In this Analysis, we combine a systematic phylogenomic survey of NAPs with quantitative mass spectrometry data on NAP abundance to uncover evolutionary drivers of chromatin diversity in archaea.

## Phylogenomic survey of NAPs in archaea

**Fig. 1:**
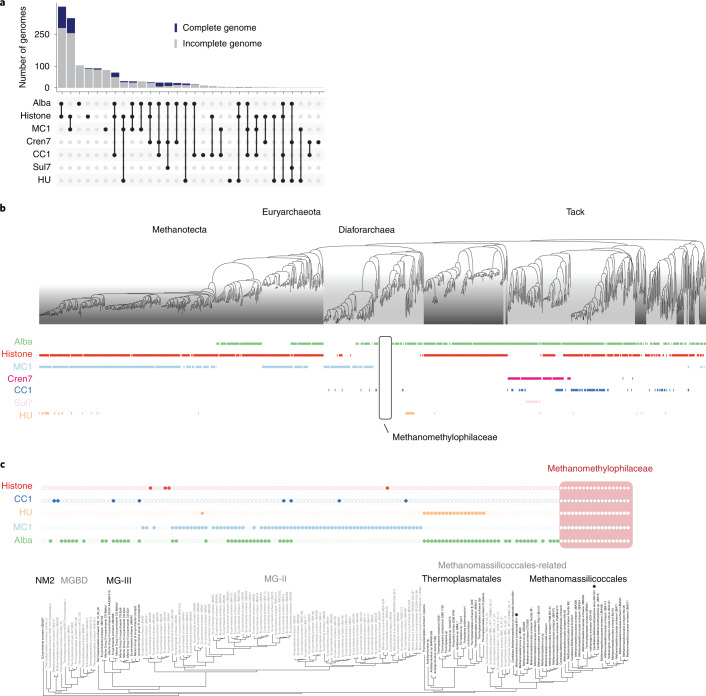
Distribution of NAPs across archaea. **a**, Co-occurrence of previously characterized archaeal NAPs in 1,419 sequenced archaeal genomes. **b**, Presence/absence of NAPs in phylogenetic context, highlighting the Methanomethylophilaceae as a family without any previously characterized NAPs. For species-level information, see Supplementary Table 1 and Supplementary Fig. 1. **c**, NAPs in the Diaforarchaea. Presence/absence of NAPs in phylogenetic context, highlighting the absence of known NAPs in the Methanomethylophilaceae, the lineage-restricted distribution of HU and MC1, and the patchy distribution of Alba. The species-level phylogeny is based on GTDB (Methods). The two Methanomassiliicoccales species for which proteomics data were generated are marked with asterisks. Species-level phylogenies are based on GTDB (Methods). Source data

To provide an up-to-date view of NAP diversity across archaea, we first collated a list of previously described archaeal NAPs (Table 1) and used hidden Markov model (HMM) scans to establish the presence/absence of NAP homologues in 1,419 archaeal genomes that represent the known archaeal diversity (Supplementary Table 1 and Methods). As highlighted previously^1^, archaeal chromatin is not dominated by a single protein but by small cliques of typically two (and sometimes three or more) abundant proteins (Fig. 1a,b and Supplementary Table 1). Different NAPs from a pan-archaeal repertoire can co-occur in most any clique, which are frequently dismantled by gene loss and absorb new members via horizontal gene transfer (HGT). Across our sample, any given NAP can be found partnering with any other (Fig. 1a and Supplementary Fig. 1), suggestive of functional promiscuity. While some NAPs are phylogenetically widespread, none is universal to archaea (Fig. 1a,b). Histones and Alba are the most common and were probably present in the last archaeal common ancestor, but both have been lost in different lineages (Fig. 1b). Conversely, gains are common and frequently driven by HGT (see below).

**Table 1:** Names and properties of previously characterized NAPs

NAP	Organism	Name	Length (amino acids)	Isoelectric point (pI)
Alba	*S. acidocaldarius*	albA	97	10.4
CC1	*T. tenax*	CC1	56	10
Cren7	*S. acidocaldarius*	creN7	59	9.99
Histone	*M. fervidus*	HMfA/B	69	9.59/8.06
HU	*T. acidophilum*	HTa	90	10.74
MC1	*M. thermophila*	MC1	93	10.32
Sul7	*S. acidocaldarius*	Sso7d	64	9.68

One clade with substantial variation in NAP repertoires is the Diaforarchaea (Fig. 1c). Both histones and *albA* have been lost at the root of this clade, but several lineages, including the Thermoplasmatales, later re-acquired *albA* from different sources, as supported by the polyphyletic distribution of diaforarchaeal homologues on a pan-archaeal Alba tree (Supplementary Fig. 2). Subsequent to *albA*/histone loss, NAP repertoires evolved in an idiosyncratic fashion along different diafoarchaeal lineages. For example, we previously described a highly expressed protein, HTa, with histone-like binding behaviour in *Thermoplasma acidophilum*^3,11^. This protein is a homologue of HU, an NAP that is widespread in bacteria but rare in archaea. HTa was probably acquired from bacteria via HGT at the root of the Thermoplasmatales; it is absent from the remainder of the Diaforarchaea (Fig. 1c). Similarly, most members of the marine group II (MG-II) archaea encode MC1, a NAP best known from *Methanosarcina* spp.^12^ and widespread among haloarchaea (Fig. 1b). Again, MC1 is present only in MG-II but absent from other diaforarchaeal lineages, and was probably acquired via HGT (Fig. 1c and Supplementary Fig. 2). Most curiously, we find that the members of one diaforarchaeal lineage, the Methanomethylophilaceae, encode no known NAPs whatsoever (Fig. 1c).

## Candidate NAPs in the Methanomassiliicoccales

**Fig. 2:**
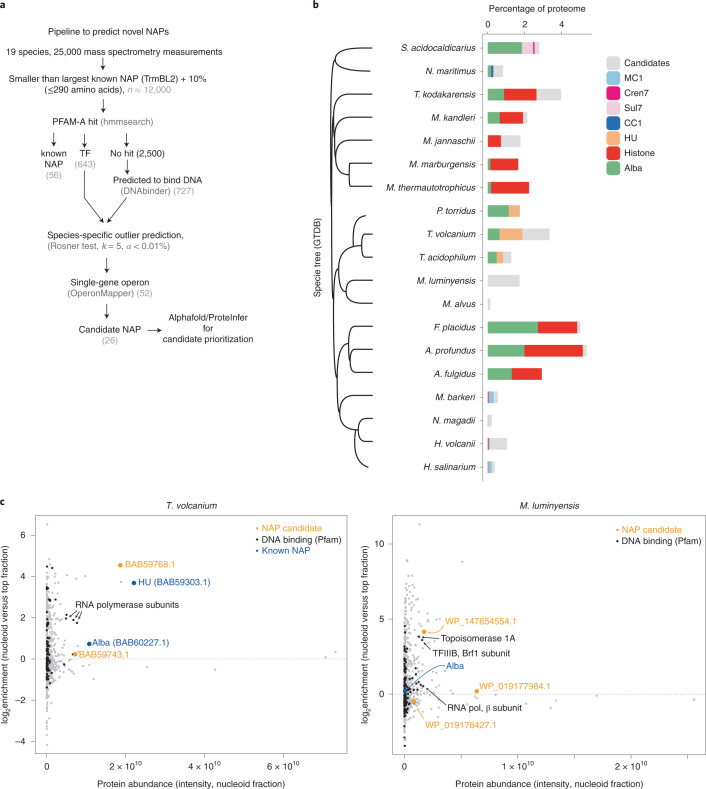
Quantitative variation in the abundance of known and predicted NAPs. **a**, Outline of the bioinformatic pipeline to predict novel NAPs. Proteins detected by mass spectrometry need to pass several successive filters to be considered as a candidate NAP. **b**, Large variation in the relative abundance (percentage of proteome) of known and candidate NAPs in 19 species of archaea for which quantitative mass spectrometry data were analysed. The species tree is taken from GTDB, with *Picrophilus torridus* and *H. salinarum* added manually. **c**, Abundance and enrichment of proteins in the nucleoid fraction in *T. volcanium* and *M. luminyensis*. Source data

Methanomethylophilaceae lack known major NAPs, but is this because they have as yet uncharacterized NAPs or do they somehow make do without NAPs? To begin to address this question, we produced quantitative mass spectrometry data for two members of the Methanomassiliicoccales, both isolated from the human gut: *Methanomassiliicoccus luminyensis*^13^ and *Methanomethylophilus alvus*. We detected and quantified 72% of the predicted proteome in *M. alvus* and 67% in *M. luminyensis*, in line with other efforts to catalogue proteins across the tree of life^6^ (Supplementary Fig. 3). *AlbA*, though present in the genome of *M. luminyensis*, was not expressed at detectable levels. We developed a bioinformatic pipeline to predict proteins that might have a role in nucleoid organization similar to known NAPs (Fig. 2a and Supplementary Methods). To qualify as a candidate NAP, proteins needed to meet four criteria. Size could not exceed that of characterized NAPs, so we considered only proteins smaller than 290 amino acids, 110% the size of TrmBL2 in *T. kodakarensis* (see below). Predicted proteins had to either contain a known DNA-binding domain or be predicted to bind DNA. Third, they needed to be expressed at a level that makes them high-abundance outliers compared with predicted transcription factors, objectively determined using Rosner tests. Fourth, they had to be encoded as single-gene operons, because known NAPs are usually present as single-gene operons (Supplementary Fig. 4).

Three proteins in *M. luminyensis* met these criteria. WP_019177984.1 is a small (74 amino acids), basic (pI: 9.64), lysine-rich protein that constitutes 1.34% of the *M. luminyensis* proteome (Fig. 2b and Supplementary Table 1), making it the 12th most highly expressed protein in our sample. Its homologue in *M. alvus* (AGI86273.1) was independently identified as the sole candidate NAP in this organism, where it is less strongly expressed (0.14% of total protein, ranking 123rd out of 1,220 proteins). Relaxing criteria on single-gene operon status did not identify additional candidates for *M. alvus*. Two additional candidates were recovered in *M. luminyensis*, but their quantitative contribution to overall NAP investment was minor (together, they make up only 0.4% of total protein and are therefore approximately three times less abundant than WP_019177984.1).

Orthologues of WP_019177984.1/AGI86273.1 are present throughout the Methanomassiliicoccales, and in several bacterial genomes, particularly in Sphingomonadales and Rhodobacterales (Supplementary Fig. 5). Monophyly of the Methanomassiliicoccales homologues suggests a single acquisition event at the root of this clade, which preceded the loss of *albA* (Fig. 1c and Supplementary Fig. 5).

To prioritize candidates for experimental follow-ups, we predicted structure and function of candidates using Alphafold2 and ProteInfer, respectively (Supplementary Methods). Two of the three candidates (WP_147654554.1 and WP_019177984.1) are predicted to function as DNA-binding proteins (Supplementary Table 1). Interestingly, our top candidate (WP_019177984.1/ AGI86273.1) is predicted to have a novel fold (Supplementary Fig. 6).

## Validating candidate Methanomassiliicoccales NAPs

To establish whether any of these candidates are associated with the nucleoid in vivo, we used sucrose gradient-based nucleoid enrichment experiments and quantitative mass spectrometry (Supplementary Methods). Briefly, we compared the relative abundance of proteins in two fractions derived from sucrose gradient centrifugation: a ‘nucleoid fraction’ that is enriched for proteins associated with the nucleoid (and is frequently also enriched for membrane proteins, which co-sediment with the nucleoid^14,15^) and a ‘top fraction’ enriched for soluble, cytosolic proteins that tend to settle at a lower density. We validated our approach using *T. volcanium* as a positive control, where, on the basis of previous work^3,11^, we expect strong nucleoid enrichment of its HU homologue (BAB59303.1). In addition, our prediction pipeline suggested the presence of two previously uncharacterized NAPs (Supplementary Table 1). Reassuringly, we find HU to be highly expressed and strongly enriched in the nucleoid (Fig. 2c). More broadly, proteins with a known DNA-binding domain (based on Pfam annotations) are significantly more enriched than proteins without such a domain (Wilcoxon test, two-sided, *P* = 0.005), suggesting that the assay succeeds in enriching for proteins associated with the nucleoid. Excitingly, one of the two candidate NAPs (BAB59768.1) is almost as abundant as HU and exhibits even stronger nucleoid enrichment, pointing to the presence of a major previously uncharacterized NAP in this species. The other candidate (BAB59743.1) is also enriched, albeit less strongly.

The results above demonstrate that the nucleoid enrichment assay can capture key features of nucleoid composition in a member of the Diaforarchaea. We therefore proceeded to apply the same protocol to *M. luminyensis*, which we chose over *M. alvus* because it autolyses in low salt (similarly to *T. volcanium*) and because we wanted to further rule out that Alba, while present in the genome, is a abundant constituent of the nucleoid in this species. Of the three candidate NAPs we had identified (see above and Supplementary Table 1), one candidate, WP_019177984.1, is found at high abundance but only nominally enriched in the nucleoid (Fig. 2c). The second candidate (WP_147654554.1) is strongly enriched (~17-fold) and also among the top 3% of most abundant proteins in the nucleoid fraction. The final candidate (WP_019176427.1), which had not been predicted as a DNA-binding protein by ProteInfer, is not enriched in the nucleoid. We further confirmed that Alba is very lowly expressed and barely enriched in the nucleoid (Fig. 2c). On the basis of these results, we suggest that the Methanomassiliicoccales encode novel NAPs whose functions and mechanisms of action remain to be elucidated. Importantly, following extensive manual scrutiny, our nucleoid enrichment experiments did not reveal obvious NAP candidates in either *M. luminyensis* or *T. volcanium* that our prediction pipeline failed to predict.

## Candidate NAPs in model archaea

We applied our prediction pipeline to 17 archaeal species (including *T. volcanium*) for which published proteome-scale quantitative mass spectrometry data were available. Quantitative inventories for 13 of these species were recently published as part of a cross-kingdom proteome survey^6^ and generated using the same protocol that we used for *M. luminyensis* and *M. alvus* (Supplementary Methods).

Starting from 22,643 proteins across 17 species, and excluding known NAPs, we retrieved 22 candidate hits (Supplementary Table 1 and Supplementary Fig. 7). Reassuringly, we recover TrmBL2, a known constituent of chromatin in *T. kodakarensis* where it is unusually abundant compared with TrmB homologues in other archaea (Supplementary Fig. 8). For some species (for example, *Methanothermobacter marburgensis* and *Archaeoglobus fulgidus*) we identified no additional candidates, suggesting that our pipeline is not excessively greedy (Fig. 2b). For others (for example, *Sulfolobus acidocaldarius*), we retrieved only candidates that are much less abundant than known NAPs in the same organism. In contrast, we also find species where novel candidates make up a substantial portion of the overall investment in NAPs, rivalling or even dwarfing the abundance of known NAPs. Notably, this list includes the model archaeon *Haloferax volcanii*, where the two candidate NAPs (HVO_1577 and HVO_2029) are considerably more abundant than either histones or MC1 (Fig. 2b), a finding we confirm in an independently generated proteomics dataset (Supplementary Fig. 9).

Intrigued by this finding, we carried out nucleoid enrichment assays in *H. volcanii* (Methods). Proteins with a Pfam DNA-binding domain are strongly enriched in the nucleoid fraction (Wilcoxon test, *P* = 2.9 × 10^−10^), suggesting that the assay worked as intended. In contrast to our findings for the Diaforarchaea, however, and despite strong ProteInfer predictions of a DNA-binding function (Supplementary Table 1), we do not find our candidates to be enriched in the nucleoid fraction (Supplementary Fig. 9), suggesting that further work is advisable to clarify their role in nucleoid biology.

## Investment in NAPs varies among archaea

As evident from the above, individual NAPs in the same organism can vary widely in abundance. We wondered whether there were also differences in NAP abundance between species. Do some species allocate substantially more of their cellular energy budget towards the production of NAPs than others? If so, what are the ecological and evolutionary drivers of differential investment in NAPs vis-à-vis other proteins? To address this question, we considered relative NAP investment in a given species as the sum of intensities attributable to all detected NAPs divided by the sum of intensities across all detected proteins (Methods). Compared in this manner, we find striking variation in NAP investment across species, ranging from 0.14% of total protein in *M. alvus* to 5.38% in *Archaeoglobus profundus* (Fig. 2b). Individual NAPs can vary over a similar range: relative histone abundance, for example, varies up to 400-fold (Supplementary Fig. 10), from 3.2% of the proteome in *A. profundus* to <0.06% in *Nitrosopumilus maritimus*, *Methanosarcina barkeri* and the Halobacteriales, where abundance is indistinguishable from that of sequence-specific transcription factors (Supplementary Fig. 10). Variable investment in NAPs is evident with or without considering candidate NAPs (Fig. 2b; range without candidate NAPs 0.03–5.16%).

We considered whether variability in NAPs is not biologically meaningful but is instead attributable to experimental factors. It is conceivable, for example, that a NAP, once detected, might represent an artificially high proportion of a proteome simply because comparatively few proteins were quantified. However, we found no significant correlation between fractional coverage of the predicted proteome and the proportion allocated to NAPs (*ρ* = −0.31, *P* = 0.19). Further, differential investment was evident when relative abundance was scaled to the abundance of house-keeping genes (transfer RNA synthetases), which show low cross-species variability (Supplementary Fig. 11 and Methods), rather than to the total proteome.

To confirm that fractional protein abundances can be compared across species, we asked whether the relative abundance of a protein in one species is usually predictive of the relative abundance of its homologue in another species. Considering reciprocal best-blast hits between species as an indicator of homology, we find this to be the case. Organisms that are phylogenetically related or ecologically close tend to have more correlated abundance profiles (Supplementary Fig. 12). This is particularly evident when, instead of considering individual reciprocal best-blast hits, we aggregate protein abundance by Pfam domain content or gene ontology category (Methods and Supplementary Fig. 12). These results indicate that quantitative comparisons across species can be made using fractional intensities as a metric. The results also advocate the use of lower granularity. Below, we therefore consider the abundance of all NAPs collectively.

## Growth temperature is correlated with NAP investment

**Fig. 3:**
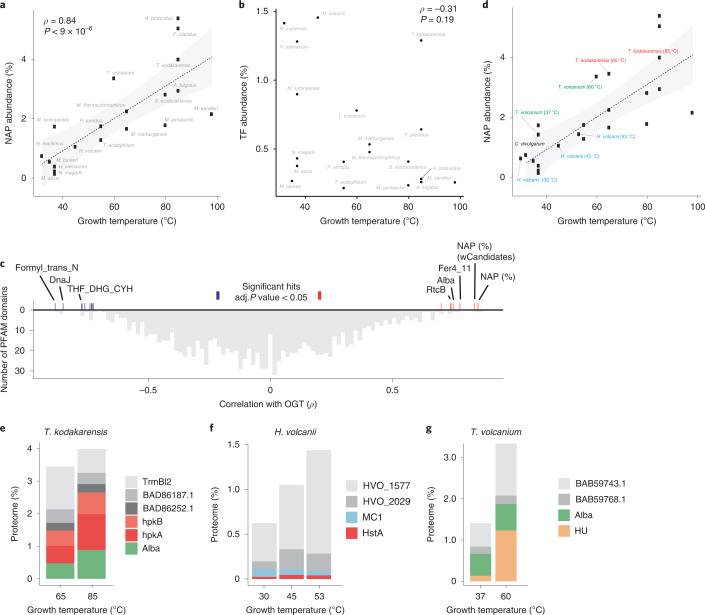
Relationship between growth temperature and NAP investment across archaea. **a**, Relationship between NAP abundance and OGT across archaea. The line of best fit for a simple linear model is shown along with 95% confidence intervals (Spearman’s *ρ* = 0.84, *P* < 9 × 10^−6^). **b**, The aggregate abundance of transcription factors (TF) is not correlated with OGT (Spearman’s *ρ* = −0.31, *P* < 0.19). **c**, Distribution of correlation coefficients between growth temperature and the relative abundance of 1,154 Pfam domains. The relative abundance of NAPs, considered as an aggregate class, exhibits the strongest correlation with growth temperature. We obtain similar results when considering gene ontology categories instead of Pfam domains. **d**, Relationship between NAP abundance and growth temperature; same as **a** but including data from *C. divulgatum* and archaea grown at different non-OGT temperatures. The regression line and 95% confidence intervals are the same as in **a**. **e**–**g**, Variability in NAP investment as a function of growth temperature in *T. kodakarensis*, *H. volcanii* and *T. volcanium*. Source data

We next asked whether relative NAP investment is a function of genome size, where organisms with larger genomes need to make a greater relative investment in NAPs because they have more DNA to manage, but this was not the case (*ρ* = −0.3, *P* = 0.21). To gain clues into potential ecological drivers of NAP investment, we identified proteins (or protein domains/functional categories) that quantitatively co-vary with NAP investment across species (Supplementary Methods). Among the most highly correlated domains, we find several that are classically associated with heat stress, including the protein chaperones Hsp20 and prefoldin but also RTCB, which has been implicated in recovery from stress-induced RNA damage^16^ (Supplementary Fig. 13). Prompted by these findings, we examined several environmental and phenotypic variables, including optimal growth temperature (OGT), pH and doubling time. We found that relative abundance of NAPs is uniquely, and strongly, associated with OGT (*ρ* = 0.83, *P* = 8 × 10^−6^; Fig. 3a and Supplementary Table 2). This finding is robust to inclusion/exclusion of candidate NAPs (Supplementary Fig. 14) and holds true for individual NAPs where these are sufficiently widespread to allow cross-species comparisons (histones, Alba; Supplementary Fig. 14). Importantly, the relationship between NAP abundance and OGT is preserved when controlling for phylogenetic non-independence (Methods).

In addition to genome size, NAP investment might be expected to scale with ploidy. As ploidy was not explicitly determined for any of the samples analysed here, we instead normalized NAP investment by investment in core transcription machinery (Supplementary Methods), which should similarly scale with ploidy. We still find a strong relationship between NAP investment and OGT (*ρ* = 0.82, *P* = 1.8 × 10^−5^), suggesting that ploidy is not a confounding factor. This is further supported by the observation that, unlike for NAPs, the relative abundance of sequence-specific transcription factors does not co-vary with temperature (Fig. 3b).

To probe whether a strong relationship between the abundance of a class of proteins and OGT is expected, we computed the correlation between OGT and the relative abundance of 1,154 Pfam domains (and 297 gene ontology categories) across the 19 species in our analysis. NAPs (considered as an aggregate class) had the strongest relationship with growth temperature (Fig. 3c).

Finally, to validate our predictions, we measured global protein abundance for *Cuniculiplasma divulgatum*, which belongs to the order Thermoplasmatales but optimally grows at 37 °C instead of 60 °C (ref. ^17^). Known/candidate NAPs were identified/predicted as described above. Assuming a linear relationship between growth temperature and NAP investment, we would expect relative NAP abundance in *C. divulgatum* of 0.75% (95% confidence interval 0.02–1.49%). We find NAP relative abundance to be 1.44%, within the predicted range (Fig. 3d).

## NAP levels vary with temperature over physiological timescales

If growth temperature is correlated with NAP abundance over evolutionary timescales, might the same be true for physiological timescales? Although no systematic data exist that span the diversity of species examined above, previous temperature shift experiments from various archaea support this hypothesis. For example, NAP abundance is affected by temperature in *T. kodakarensis*^18^, with reduced levels of histones and Alba driving a 13.5% relative drop in total NAP investment at 65 °C compared with 85 °C (Fig. 3e). Similarly, levels of NAP investment in *H. volcanii* increase and decrease, respectively, when growth occurs at temperatures above (53 °C) or below (30 °C) the OGT (45 °C)^19^, although we note that this holds only when candidate NAPs (in particular, HVO_1577) are included (Fig. 3f). Where we lack protein-level data, RNA dynamics paint a similar picture: histone transcripts are downregulated upon cold shock in *Methanococcus jannaschii*^20^, as are histones and *mc1* in *Methanococcoides burtonii*^21^, while *sul7* expression increases upon heat shock in *Sulfolobus solfataricus*^22^.

To provide further independent support for a physiological relationship between NAP investment and temperature, we cultured *T. volcanium* at 37 °C, and determined global protein abundances as described above. In general, protein abundances are similar at 37 °C and 60 °C (Supplementary Fig. 15). However, NAP abundance is reduced by more than 50%, from 3.36% of the protein budget at 60 °C to 1.42% at 37 °C (Fig. 3d), mainly owing to decreased amounts of HU (Supplementary Fig. 15).

## Discussion

Selection for increased thermostability has left conspicuous footprints on the composition of proteins and RNAs in many species. Proteins from thermophiles are, for example, enriched in charged and hydrophobic amino acids while their structural RNAs (tRNAs and ribosomal RNAs) exhibit higher-than-average GC content, consistent with the need for stronger base-pair bonds at higher temperatures^23,24^. Similar compositional hard-coding was also hypothesized to occur at the DNA level, where it was proposed that thermophiles would have genomes with increased GC content, but this was not the case^23^. *T. kodakarensis* (52% GC, OGT 85 °C) and *Pyrococcus furiosus* (41% GC, OGT 100 °C) show that average genomic GC content is compatible with growth at high temperatures^25^. Here we find that increases in OGT are associated with differential investment in NAPs in archaea.

Several in vitro studies on archaeal histones^26,27^, Sul7 (refs. ^28,29^), HTa^30,31^ and MC1 (ref. ^12^) have shown that NAP binding can increase DNA melting temperature, reduce the risk of DNA denaturation and/or promote strand re-annealing, which is relevant during both accidental and programmed opening events that occur in transcription, replication and repair. Our data are consistent with a model in which the risk of denaturation, which increases with temperature, underpins differential investment in NAPs across archaea. This could be explored further in the future by, for example, measuring denaturation in vivo by quantifying the amount of single-stranded DNA at different temperatures in wild type and NAP deletion mutants.

Promoters, which are AT rich and open to enable transcription, are hotspots for denaturation at higher temperatures. Thermophiles seem to have reduced potential death due to promoter-initiated denaturation in part by having a reduced set of promoters compared with mesophiles: the number of genes per transcription unit, co-expressed from a single upstream promoter, increases with temperature (Supplementary Fig. 16). In addition, the proportion of the genome dedicated to intergenic regions decreases with temperature in archaea^32^. Pinning the promoter on either side with DNA-binding proteins—as observed for histones^33^ but also HTa in *T. acidophilum*^3^—might have evolved in parallel to prevent uncoordinated promoter melting and runaway extension of the resulting denaturation bubbles.

Protection from denaturation as a function of NAPs is also consistent with the diversity of NAPs in archaeal genomes, epitomized by the Diaforarchaea. Proteins with various folds can bind to DNA and thereby raise its melting temperature and curb denaturation. Our model of NAP evolution is consistent with the lack of co-variation of NAP abundance with the abundance of other chromatin factors during evolution (based on correlations between Pfam domains, as in Fig. 3c) and with the observation that NAPs are usually encoded as single-gene operons. Both observations suggest a scarcity of functional dependencies.

A limitation of our analysis is that it cannot exclude specific adaptive roles that might have selected for NAP diversity in archaea. One such adaptive role might be in the prevention, detection and repair of DNA damage^25^. Mutagenic challenges differ across environments and might favour some NAPs over others. MC1, for example, protects against radiation damage^34^, a frequent insult for halophiles that live in shallow aquatic environments. Conversely, Cren7 binds to T:G mismatches produced by cytosine deamination events^35^, which become more common at higher temperature. We propose that, while denaturation might shape total NAP abundance, NAP diversity is probably a product of both exchangeability and species-specific requirements for nucleoid function and maintenance. This model of NAP evolution can be tested in the future with deletion-and-rescue experiments to determine which NAPs can complement the loss of which other.

Chromatin components can be acquired from other archaea or bacteria, as illustrated in Diaforarchaea where HTa, Alba, MC1 and the newly identified Methanomassiliicoccales protein can trace their origin to horizontal transfers. In addition, components can arise from repurposing of proteins already present in the proteome, as transcription factors, like TrmBL2 in *T. kodakarensis*, become global chromatin constituents. Conversely, proteins can lose their global architectural roles. In the most extreme case, abundant NAPs have been completely lost in the Methanomethylophilaceae. They can also undergo significant reductions in abundance. This is what seems to have happened to histones in halophiles and other lineages, consistent with their non-essential status in *H. salinarum*^7^ and *Methanosarcina mazei*^36^. The low abundance of HstA in *H. volcanii*, at both the transcript^5^ and protein level (Figs. 2b and 3f), is hard to reconcile with its purported role as a major architectural factor^37^. We therefore suggest that previous findings of widespread protection from micrococcal nuclease digestion in this species might, in fact, be caused not by histones but by an as yet uncharacterized protein or set of proteins. Our de novo prediction pipeline suggests HVO_1577, a protein that contains an HrcA DNA-binding domain (Supplementary Fig. 7), as a candidate that deserves further investigation.

Our data show that, when moving from a thermophilic to a mesophilic niche, different lineages of archaea have reduced their investment in NAPs. We do not think that this means that all archaeal mesophiles have reduced investments in chromatin. For example, histones are highly expressed, at least at the transcript level, in some mesophilic members of the Methanobacteriales, notably *Methanobrevibacter smithii*, which grows at 37 °C (its three histones are ranked 1st, 8th and 282nd most highly expressed^5^). Whether this also holds true at the protein level remains to be established, but we suggest that high levels of chromatinization might be obligatory for thermophiles but facultative for mesophiles.

Finally, eukaryotes are mainly mesophiles, but their DNA is ubiquitously wrapped in nucleosomes, and removal of histones results in uncontrolled gene expression^38^. The acquisition of histone tails, possible in the lineage of archaea, the Asgardarchaea, from which eukaryotes are thought to have emerged^39^, and their subsequent use for signalling might have been one of the factors driving entrenchment, generating a thick top layer of cellular machinery that acts on, modifies and remodels nucleosomes to orchestrate gene expression, DNA repair and replication. Over time, the evolution of cryptic promoters, rendered inaccessible by nucleosomes but activated following their removal, might also have contributed to the retention of global chromatinization^38^.

Irrespective of the factors that first rendered eukaryotic histones indispensable, we speculate that high levels of chromatinization in eukaryotes might represent an evolutionary relic of thermophilic ancestry, and that eukaryotes—unlike many archaea—evolved a dependency on global chromatinization that they were unable to break when adapting to a more temperate niche.

## Methods

### Strain and growth conditions

*M. luminyensis* and *T. volcanium* were obtained from DSMZ (DSM 25720), and *M. alvus* (isolate Mx-05) had been isolated previously by one of us (J.F.B.). *H. volcanii* (strain H28) and *C. divulgatum* (strain S5(T)) were kind gifts from Thorsten Allers and Olga Golyshina, respectively. Both Methanomassiliicoccales strains were grown under strictly anaerobic conditions (2 atm. of H_2_/CO_2_ 20%/80%) using 10 ml of growth medium in 50 ml glass bottles, sparging the head space, and maintained in an anaerobic growth chamber according to DSMZ recommendations for *M. luminyensis* with one exception: ruminal fluid (200 µl) was added for *M. alvus*. Cultures were incubated without shaking at 37 °C using 60 mM of methanol as the electron acceptor for methanogenesis. *M. luminyensis* cultures were transferred from their culture vials to collection tubes in the anaerobic growth chamber. *T. volcanium* was grown as recommended by DSMZ, and *H. volcanii*^40^ and *C. divulgatum* as previously described^17^.

### Cell pellet preparation for whole cell extracts

Fifty-millilitre aliquots of 10 day (3 day) cultures of *M. luminyensis* (*M. alvus*) were pelleted under anaerobic conditions and stored at −80 °C. 0.5 (0.9) optical density equivalent to 4 (3) days cultures grown at 37 °C of *C. divulgatum* (*T. volcanium*) (shaking at 180 rpm in an Infors incubator) were pelleted, resuspended in neutralized (pH 4) pre-warmed medium before pelleting and storage at −80 °C. Protein pellets were prepared following PreOmics iST kit guidelines.

### Protein extraction, preparation and mass spectrometry

For whole cell extracts, ~10 mg of frozen pellet was resuspended in lysis buffer and volumes were adjusted after the heating step to load 100 µg as measured by nanodrop absorbance at 205 nm. For nucleoid enrichment experiments, volumes were adjusted after the heating step so that loaded material was similar for top and nucleoid fractions, which ranged from 30 µg to 100 µg depending on the species of interest. As indicated by the manufacturer, samples were heated for 10 min at 95 °C in the lysis buffer. The heating step was extended to 1 h for *H. volcanii* nucleoid enrichment samples to reverse cross-linking (see below). Following the manufacturer’s instruction, a DNA sonication step was included (Diagenode Bioruptor, ten cycles; 30 s ON/OFF, high intensity) followed by digestion (using the PreOmics iST kit Trypsin/LysC mix) for 1.5 h at 37 °C with shaking. The whole procedure was carried out without interruption, and pellets were stored at −80 °C in MS-LOAD buffer before being processed by mass spectrometry (two biological replicates with technical replicates for each, except for *M. luminyensis* nucleoid enrichment, which was done in biological triplicates).

### Liquid chromatography–tandem mass spectrometry analysis

Samples were injected in technical duplicates in either nano-flow or micro-flow modes.

For nano-flow analysis, chromatographic separation was performed using an Ultimate 3000 RSLC nano liquid chromatography system (Thermo Scientific) coupled to an Orbitrap Q-Exactive mass spectrometer (Thermo Scientific) via an EASY-Spray source. Peptide solutions were injected and loaded onto a trapping column (Acclaim PepMap 100 C18, 100 μm × 2 cm) for desalting and concentration at 8 μl min^−1^ in 2% acetonitrile, 0.1% TFA. Peptides were then eluted on-line to an analytical column (EASY-Spray PepMap RSLC C18, 75 μm × 75 cm) at a flow rate of 200 nl min^−1^. Peptides were separated using a 120 min gradient, 4–25% of buffer B for 90 min followed by 25–45% buffer B for another 30 min (composition of buffer B: 80% acetonitrile and 0.1% FA) and subsequent column conditioning and equilibration. Eluted peptides were analysed by mass spectrometry in positive polarity using a data-dependent acquisition mode. Ions for fragmentation were determined from an initial MS1 survey scan at 70,000 resolution, followed by higher-energy collision-induced dissociation of the top 12 most abundant ions at 17,500 resolution. MS1 and MS2 scan AGC targets were set to 3 × 10^6^ and 5 × 10^4^ for maximum injection times of 50 ms and 50 ms, respectively. A survey scan *m*/*z* range of 400–1,800 was used, normalized collision energy set to 27 and charge exclusion enabled for unassigned and +1 ions. Dynamic exclusion was set to 45 s.

For samples run in micro-flow, chromatographic separation was performed using an Ultimate 3000 RSLC nano liquid chromatography system (Thermo Scientific) coupled to an Orbitrap Q-Exactive mass spectrometer (Thermo Scientific) via an EASY-Spray source. Peptide solutions were injected directly onto the analytical column (Waters nanoEase M/Z Peptide CSH C18, 300 μm × 15 cm) at working flow rate of 5 μl min^−1^ for 4 min. Peptides were then separated using a 121 min stepped gradient: 0–4% of buffer B for 11 min, 4–47.5% of buffer B for 114 min (composition of buffer A—95/5%: H_2_O/DMSO + 0.1% FA; buffer B—75/20/5% MeCN/H_2_O/DMSO + 0.1% FA), followed by column conditioning and equilibration. Eluted peptides were analysed by mass spectrometry in positive polarity using a data-dependent acquisition mode. Ions for fragmentation were determined from an initial MS1 survey scan at 70,000 resolution, followed by higher-energy collision-induced dissociation of the top ten most abundant ions at 17,500 resolution. MS1 and MS2 scan AGC targets were set to 3 × 10^6^ and 1 × 10^5^ for maximum injection times of 50 ms and 100 ms, respectively. A survey scan *m*/*z* range of 400–1,800 was used, normalized collision energy set to 27 and charge exclusion enabled for unassigned and +1 ions. Dynamic exclusion was set to 45 s.

### Raw mass spectrometry data processing

Data were processed using the MaxQuant software platform (v1.6.10.43)^41^, with database searches carried out by the in-built Andromeda search engine against various organism-specific databases from the National Center for Biotechnology Information (NCBI) GenBank. A reverse decoy database approach was used at a 1% false discovery rate for peptide spectrum matches. Search parameters were as follows: maximum missed cleavages set to 2, fixed modification of cysteine carbamidomethylation and variable modifications of methionine oxidation, protein N-terminal acetylation, asparagine de-amidation and cyclization of glutamine to pyro-glutamate. Label-free quantification (LFQ) was enabled with a LFQ minimum ratio count of 1. The ‘Match between runs’ function was used with match and alignment time limits of 0.7 min and 20 min, respectively.

### Nucleoid enrichment assays and analysis

Starting material was adjusted for each species (50 ml of exponentially growing culture, 18 h after inoculation at 60 °C for *T. volcanium*; 50 ml of exponentially growing culture, 24 h after inoculation at 45 °C for *H. volcanii*; 150 ml of a 7 day cultures, grown at 37 °C, for *M. luminyensis*). Samples were unfixed, except for *H. volcanii* (15 min 1% paraformaldehyde fixation in growth medium at growth temperature, quenched using a final concentration of 15 mM glycine for 5 min). The rationale for fixing *H. volcanii* cells was to prevent artefacts due to the difference between its high internal salt concentration and the relatively lower osmolarity of the buffer used. We followed a protocol described previously^42^, with the following modifications: frozen pellets were resuspended in low-salt buffer (500 µl 10 mM Tris 5 mM EDTA instead of buffer A) over the course of 15 min on ice. Manual homogenization was carried using a Dounce homogenizer for *T. volcanium*. Lysozyme was omitted from buffer B as we chose species that autolyse in low-salt buffers. Sucrose gradients were made in a step-wise fashion with 10% sucrose increments, 2 ml by increment and 10 ml of total volume. Gradients were allowed to cool down for at least 2 h at 4 °C before use. Samples were spun in a Beckman Optima ultracentrifuge, rotor SW 41 Ti, at 17,100*g* at 4 °C for 30 min, using the lowest acceleration and deceleration settings. Following centrifugation, 150 µl aliquots were sampled from the fraction of interest (either the very top fraction or the opaque nucleoid fraction). Proteins were precipitated using methanol and chloroform. Following maxQuant quantification, differential abundance of proteins was computed using the R package DEP^43^, using the top fraction as control.

### Genomes database

All genomes and proteomes were obtained from NCBI (https://www.ncbi.nlm.nih.gov/assembly) accessed on 21 May 2021. Proteomes that were not available from NCBI were predicted from genome sequence using Prodigal v2.6.3 with default parameters.

### Species tree and taxonomy

The archaeal species tree and taxonomic groups were obtained from the Genome Taxonomy Database (GTDB; https://gtdb.ecogenomic.org) accessed on 23 September 2020, with some species names updated to reflect current use in the literature (Supplementary Table 1).

### Processing of public proteomics data

We included only proteomes that were (1) derived from whole cell extract, (2) without size selection and (3) comprised more than 500 identified proteins. Data for *H. volcanii* was obtained from ref. ^19^ and, for Supplementary Fig. 9, from ref. ^44^, *T. kodakarensis* from ref. ^18^, *Natrialba* *magadii* from ref. ^45^ and *Nitrosopumilus* *maritimus* from ref. ^46^. Data for all other species were obtained from ref. ^6^. For each dataset, measurements that did not correspond to the Genbank complete genome of the strain/species were discarded. Correspondence between Uniprot and Genbank ID was established using the Uniprot Retrieve/ID mapping tool. For each dataset, normalized intensity (in %) was computed as the ratio of each protein intensity over the total intensity for all quantified proteins in a given species. For proteomes where this information was available, LFQ intensities instead of raw intensities were used. We confirmed that use of raw instead of LFQ intensities did not qualitatively affect conclusions.

### Protein annotations

HMM models were downloaded from PFAM (PFAM-A, accessed on 20 January 2020) and TIGR (TIGRFAMs 15.0, accessed on 15 May 2020), and sequences were searched using hmmsearch (version 3.1b2). All searches were carried out using the gathering thresholds provided for each models (option -cut_ga) to ensure reproducibility. No further threshold was applied unless mentioned otherwise. Results were robust to application of an alternative, stricter threshold of 1 × 10^−3^. As no HMM model existed for Cc1, we searched for sequences homologous to *Thermoproteus tenax* Cc1 (Uniprot ID G4RKF6) using jackhmmer (version 3.1b2), applying an e-value threshold of 1 × 10^−5^. A list of DNA-binding protein PFAM domains was obtained from ref. ^47^. In addition, all the PFAM domains contained in the PFAM2GO category 0003700 were considered in the final set of annotations of transcription factors. For normalizations, RNA polymerase abundance was taken to be the summed abundance of proteins containing the RNA_pol_Rpb1_3 PFAM domain. Similar results were obtained using the RNA_pol_Rpb2_3 HMM model. tRNA synthetase abundance was computed as the sum of all proteins having a tRNA-synth_1 or tRNA-synth_2 PFAM domain. Detailed tables and full sequences of all NAPs and candidate NAPs discussed in this study are available as supplementary material (Supplementary Table 1).

### Gene ontology

Gene ontologies were obtained from pfam2go tables, available at http://current.geneontology.org/ontology/external2go/pfam2go.

### Habitat and phenotypes

Phenotypic data were obtained from ref. ^48^ and habitat data from ref. ^49^. OGTs were obtained from bacdive-DSMZ (https://bacdive.dsmz.de/).

### Operon prediction

Operons were predicted using Operon Mapper (https://biocomputo.ibt.unam.mx/operon_mapper/) with default settings.

### DNA binding prediction

DNA binding was predicted using DNAbinder (https://webs.iiitd.edu.in/raghava/dnabinder/) using the support vector machine model trained on a realistic dataset^50^. Proteins whose score was higher than 0 were considered as possible DNA-binding proteins. Protein structures of NAP candidates were predicted as homodimers using Colabfold^51^, and functional inference was carried out using the ProteInfer webserver^52^. Similar folds were found using FoldSeek^53^ with default settings.

### Protein alignments and phylogenetic trees

Proteins sequences were aligned using MAFFT (option -linsi). With the exception of the species tree (see above), all trees were built using RAXML-NG, model LG + R6. Best maximum likelihood midpoint rooted trees are shown along with the results of 100 non-parametric bootstraps. Trees were visualized using iTol (https://itol.embl.de/).

### Phylogenetic linear regression

To control for phylogenetic non-independence, phylogenetic linear regression was carried out using the R package phylolm, Model ‘BM’ with 10,000 bootstraps or ‘OUrandomroot’. Variables were log transformed before regression.

### Reporting summary

Further information on research design is available in the Nature Research Reporting Summary linked to this article.

## Supplementary information

Supplementary Information Supplementary Figs. 1–16.

Reporting Summary

Supplementary Table Contains Supplementary Tables 1 and 2 in a single file.

Supplementary Data Contains source data for supplementary figures.

## Data Availability

All data generated or analysed in this manuscript is publicly available as follows: OGTs were obtained from bacdive-DSMZ (https://bacdive.dsmz.de/). Genomes and predicted proteomes were obtained from https://www.ncbi.nlm.nih.gov/assembly. HMM models were downloaded from Pfam and TIGRFAMs 15.0. Gene ontologies were obtained from http://current.geneontology.org/ontology/external2go/pfam2go. Archaeal trees were obtained from https://gtdb.ecogenomic.org. Mass spectrometry data generated as part of this study have been deposited in the PRIDE repository with accession code PXD034568 (https://www.ebi.ac.uk/pride/archive/projects/PXD034568/). Previously published data that were re-analysed here and support the findings of this study are available as detailed in the original publications. Source data are provided with this paper.

## Source data

Source Data Fig. 1 Contains source data to reproduce Fig. 1b.
Source Data Fig. 2 Contains source data to reproduce Fig. 2b,c.
Source Data Fig. 3 Contains source data to reproduce Fig. 3.

## Code availability

Custom code used to produce the results presented here is available at https://github.com/hocherantoine/NAPsQuant.
