# Supplementary material for: Growth temperature and chromatinization in archaea
Source: Nat Microbiol. 2022 Oct 20;7(11):1932–42. doi: 10.1038/s41564-022-01245-2 (PMC7613761; doi:10.1038/s41564-022-01245-2)
Supplement: Supplementary file 1 — Supplementary Figs. 1–16. [file 41564_2022_1245_MOESM1_ESM.pdf]

nature microbiology

Analysis

https://doi.org/10.1038/s41564-022-01245-2

Growth temperature and chromatinization in archaea

In the format provided by the authors and unedited

Supplementary Material

for

"Growth temperature and chromatinization in archaea"

(Hocher *et al.* 2022)

This supplement contains Figures S1-16

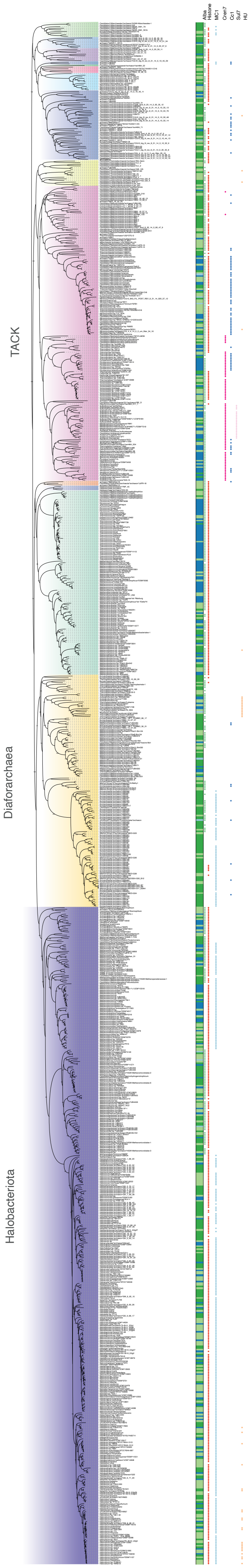
Alba
Histone
MC1
Cren7
Cc1
Sul7
HU
TACK
Diaforarchaea
Halobacteriota

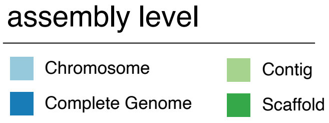
assembly level
Chromosome
Complete Genome
Contig
Scaffold

B

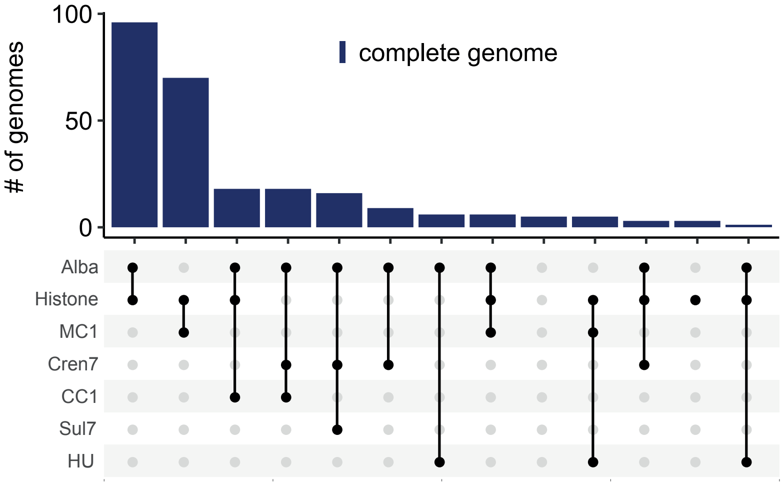
# of genomes
100
50
0
complete genome
Alba
Histone
MC1
Cren7
CC1
Sul7
HU

Figure S1. Distribution of nucleoid-associated proteins (NAPs) across the archaea. (**A**) Name and assembly level are provided for each species in phylogenetic context. The species-level phylogeny is based on GTDB (see Methods). (**B**) Co-occurrence of NAPs. This panel is equivalent to Fig 1A but only considers co-occurrence in complete genomes. Information on assembly level is based on metadata from NCBI.

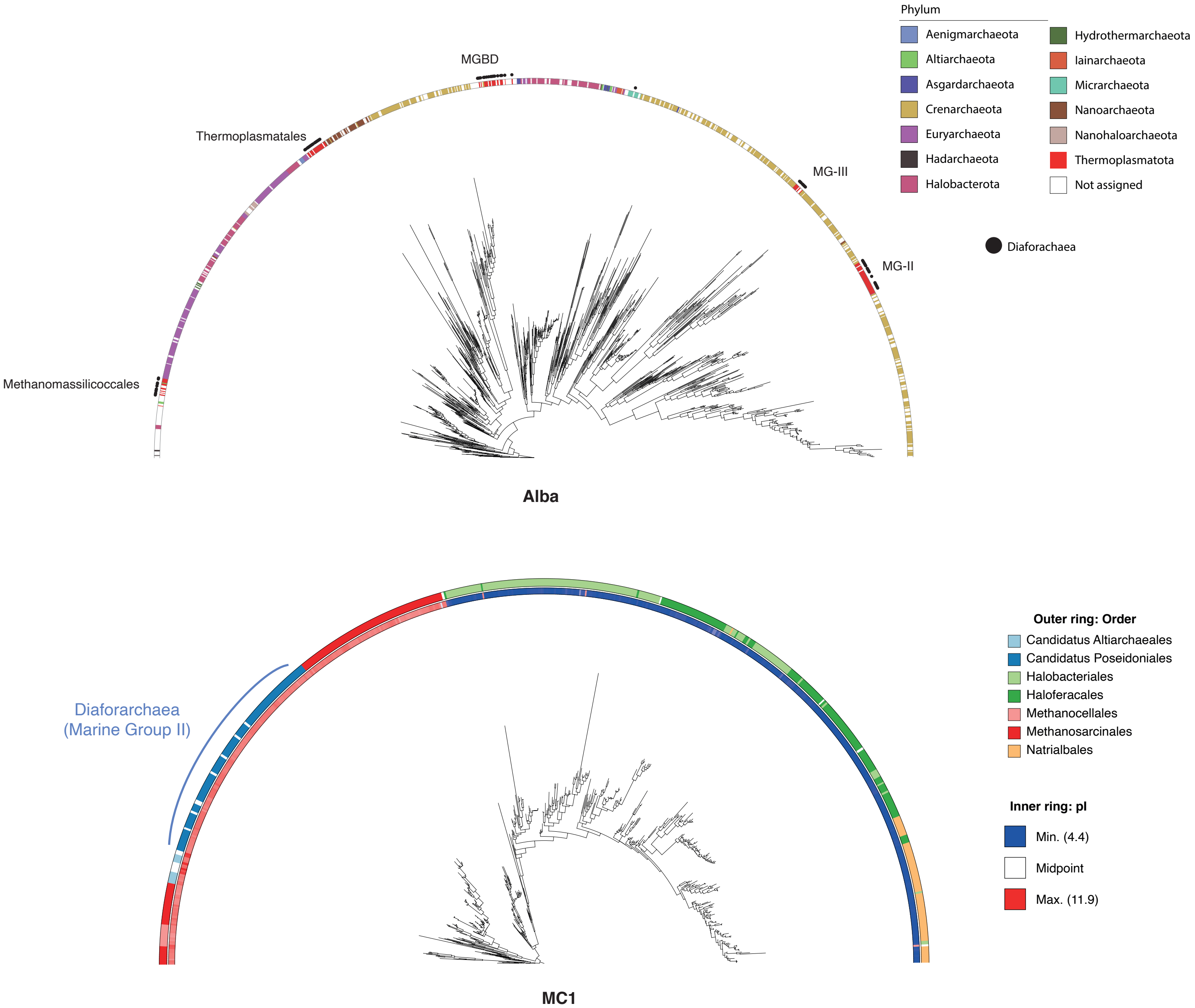

Figure S2. Maximum likelihood phylogeny of archaeal (**A**) Alba and (**B**) MC1 homologs. Alba sequences from the Diaforarchaea are not monophyletic, suggesting multiple independent acquisition events. MC1 sequences found in the Diaforarchaea are restricted to marine group II archaea and branch as a monophyletic group within the Methanosarcinales consistent with a single horizontal transfer event. Note also that sequences from haloarchaea have dramatically lower isoelectric points, making them unlikely donors. Black circles around the outside of the Alba tree highlight Alba homologs from the Diaforarchaea. MG-II (III): Marine group II (III); NM2: New Methanogen lineage 2; MGBD: Marine benthic group D.

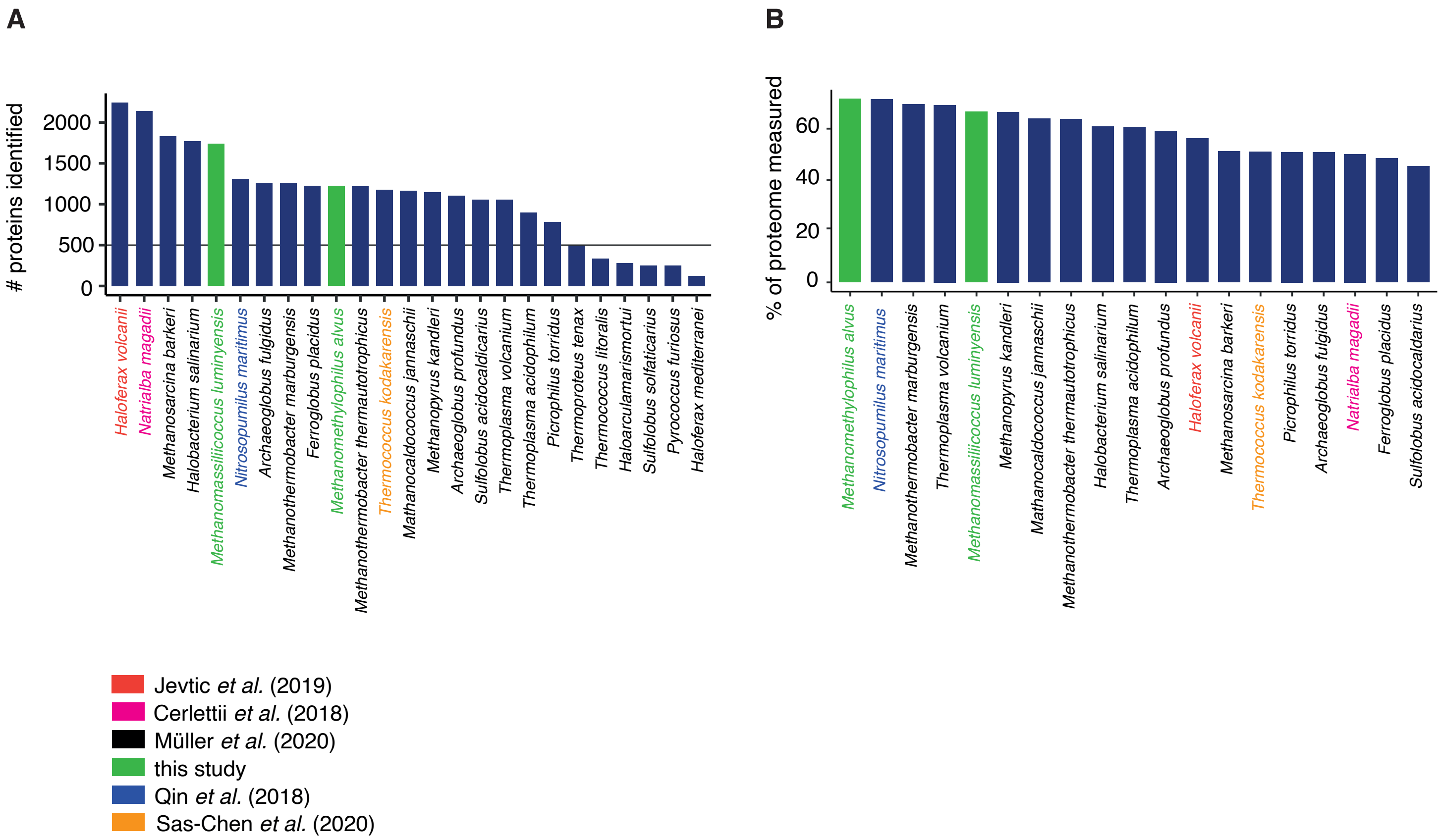

Figure S3. Absolute (**A**) and relative (**B**) coverage of the predicted proteome in 19 archaea.

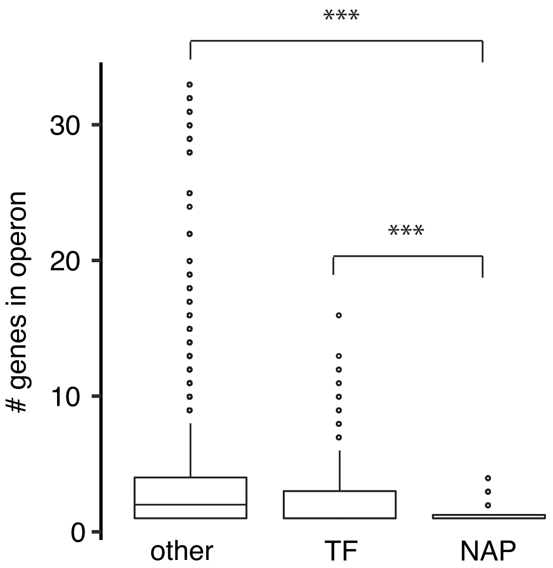

Figure S4. Sizes (number of genes) for operons that contain proteins classified as NAPs (n=56 proteins), transcription factors (TF, n=791 proteins) or “other” domains (neither NAP nor TF, n=24644 proteins) based on Pfam annotations. ***P<0.001. Two-sided Mann Whitney U test. Box plots show the median as the centre measure, the box extends to the interquartile ranges, and the whiskers to the 95th percentile.

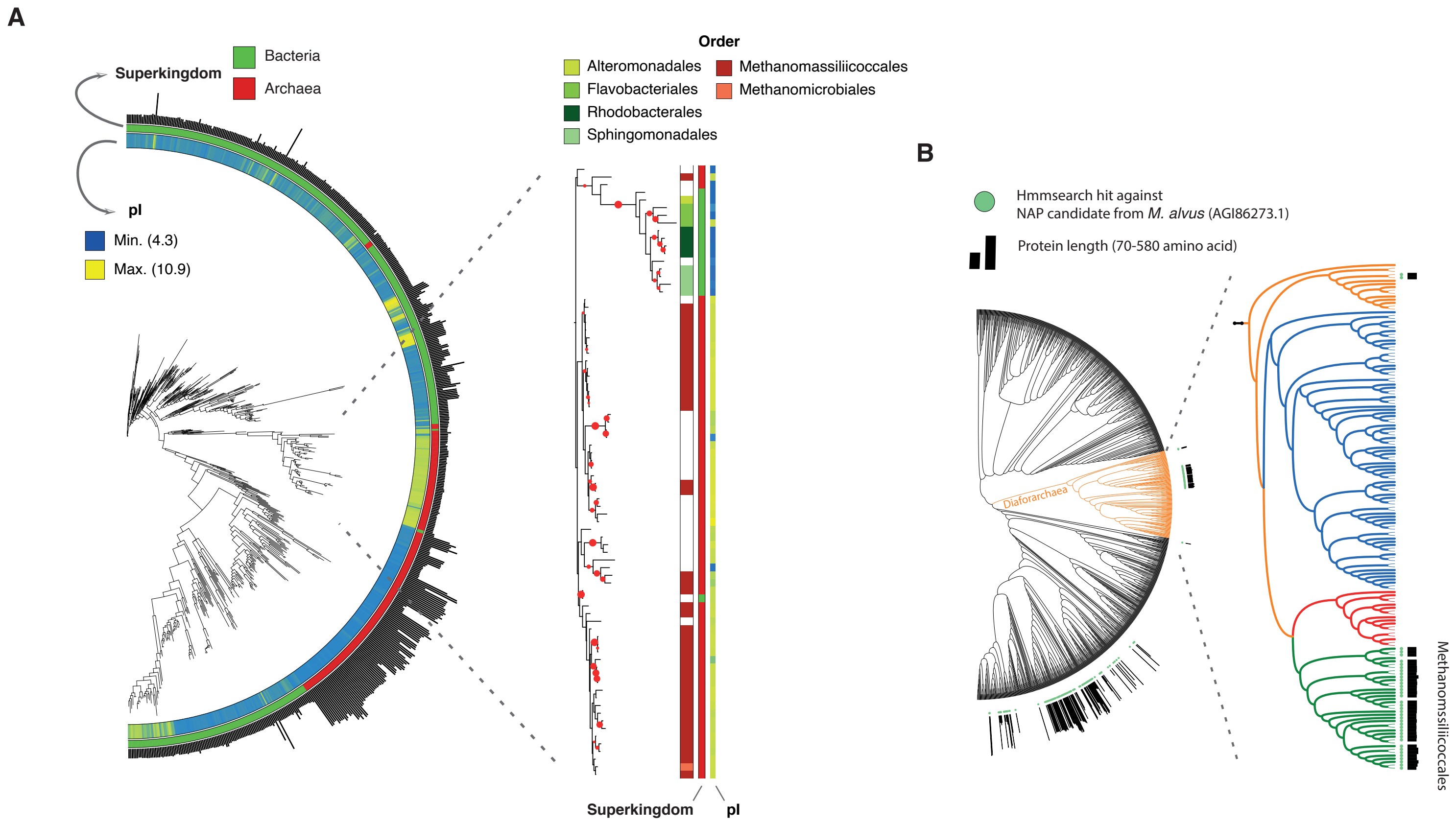

Figure S5. The novel candidate NAP WP_019177984.1/AGI86273.1 in phylogenetic context. (**A**) Maximum likelihood phylogeny of bacterial and archaeal WP_019177984.1/AGI86273.1 homologs. Homologs were obtained by running jackhmm using AGI86273.1 as a seed (e-value 1e-5). The subtree indicated by dashed lines was re-aligned and a tree computed using RAxML-NG. Bootstrap (n=100) values superior to 50 are shown. (**B**) Distribution of jackhmm hits using AGI86273.1 as a seed across archaea, displayed on a species tree obtained from GTDB.

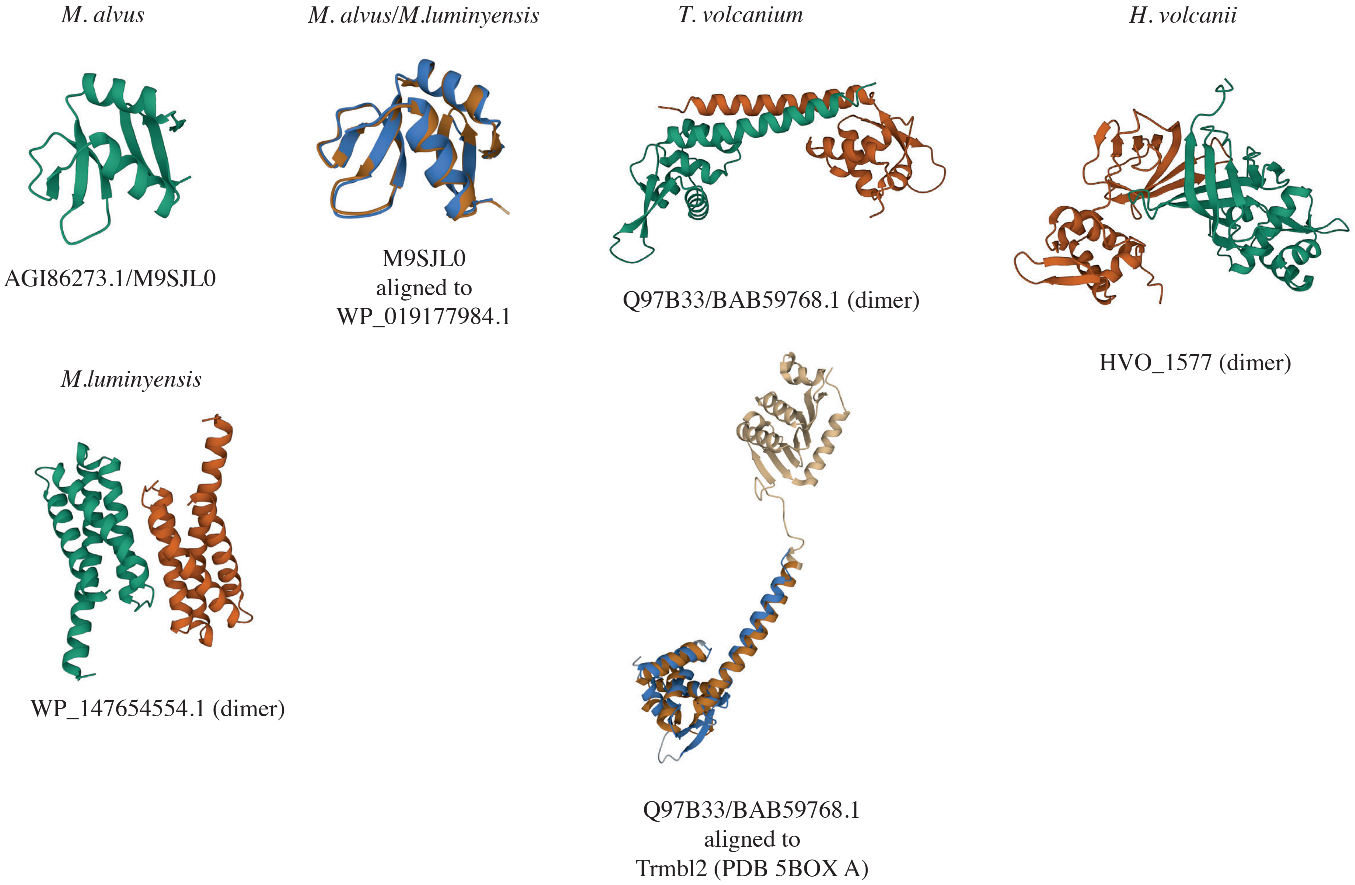

Figure S6. Alphafold-predicted structures of NAP candidates. Some structures are modelled as dimers but might not form dimers *in vivo*.

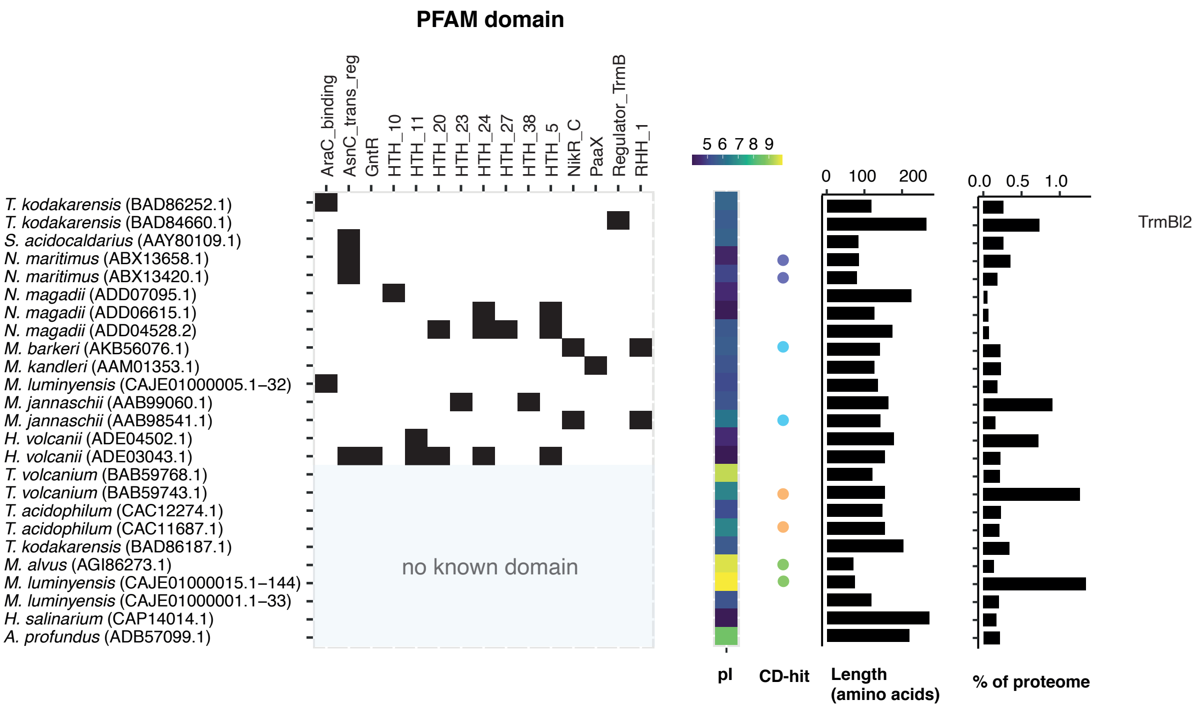

Figure S7. Overview of candidate NAPs, their domain content, size, isoelectric point (pI) and fractional abundance in the proteome of the species in which they were identified. Likely orthologs amongst this set were identified using CD-hit (see Methods) and bear the same colour. The pipeline used to predict novel candidate NAPs is described in the main text and Methods. See Table S1 for further details, including likely false positives.

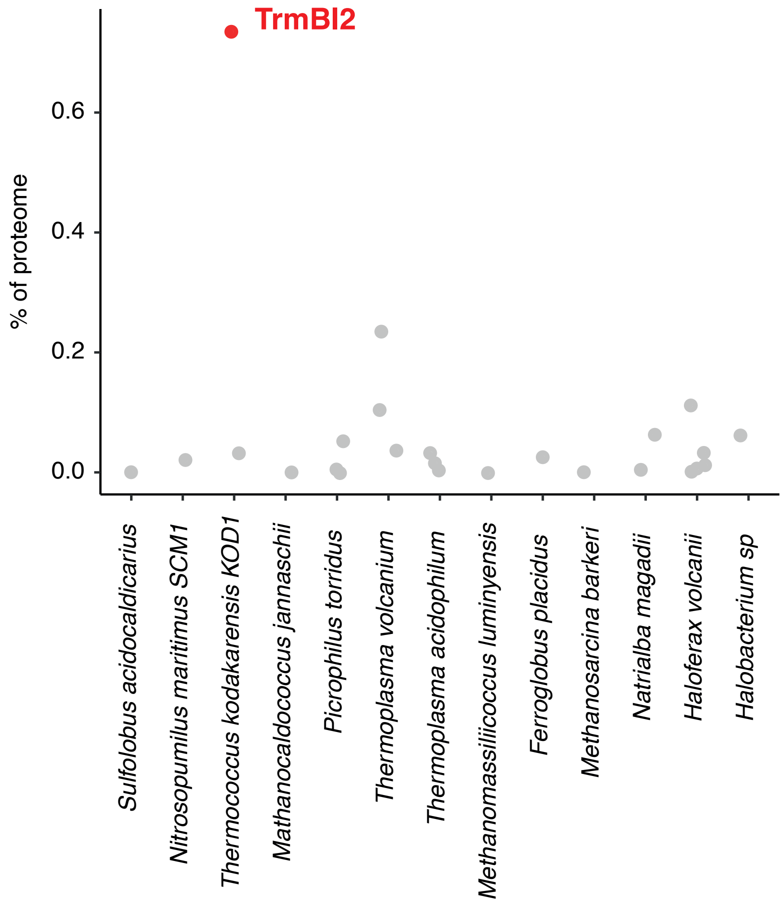

Figure S8. Relative abundance of TrmBL orthologs in different proteomes. *T. kodakarensis* TrmBL2 stands out as having a much higher relative abundance than its homologs in other proteomes.

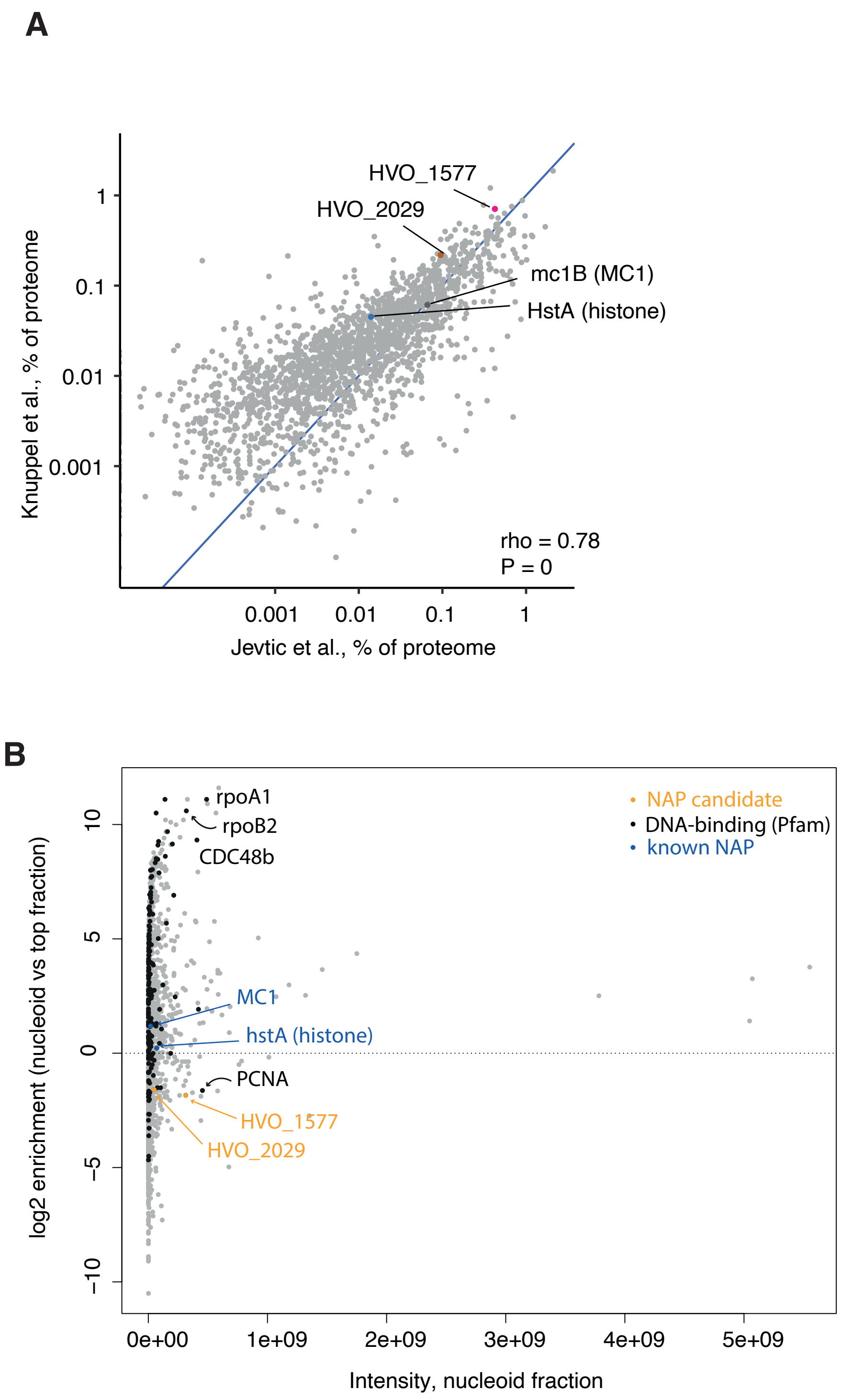

Figure S9. (**A**) Relative abundance of known and candidate NAPs in *Haloferax volcanii*, as determined in two independent quantitative proteomics studies (Jevtić et al. 2019; Knüppel et al. 2021). Note that the single *H. volcanii* histone (HstA) and MC1 are >10-fold less abundant than the novel NAP candidate HVO_1577 (Spearman's rho=0.78, P=0, N=2030 proteins detected across both studies). (**B**) Abundance and enrichment of proteins in the nucleoid fraction in *H. volcanii*.

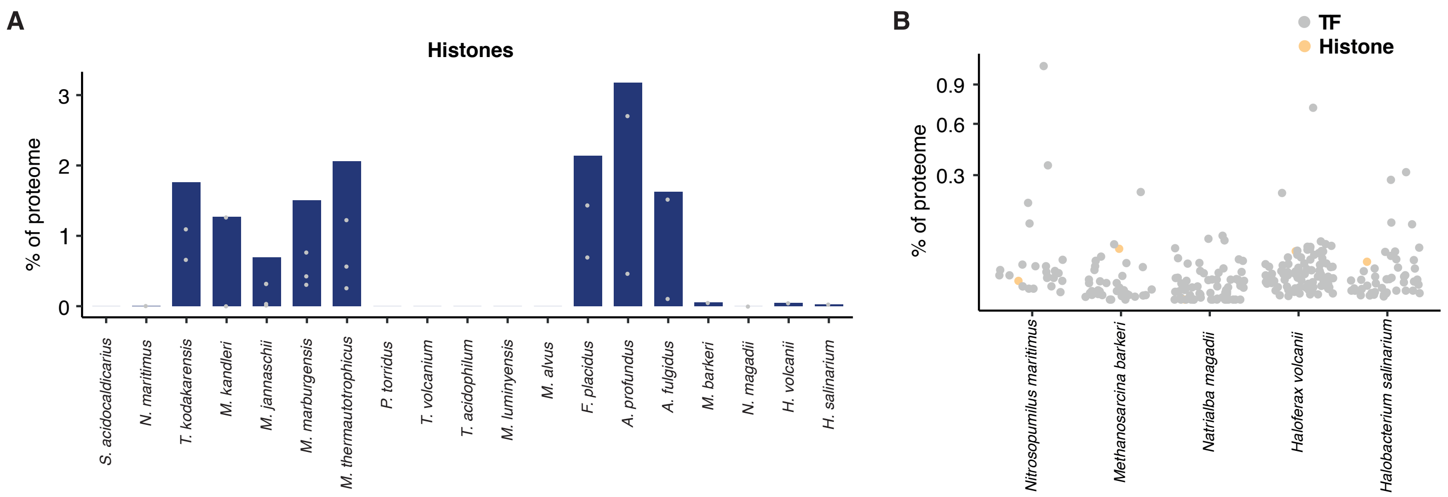

Figure S10. Quantitative variation of histone abundance across 19 archaea. (**A**) Bar heights represent the summed abundance of all detected histone paralogs in a given species, while the grey dots mark the abundance of individual paralogs. (**B**) The relative abundance of histone proteins in halophilic archaea compared to the abundance of transcription factors (TF) measured in the same experiment.

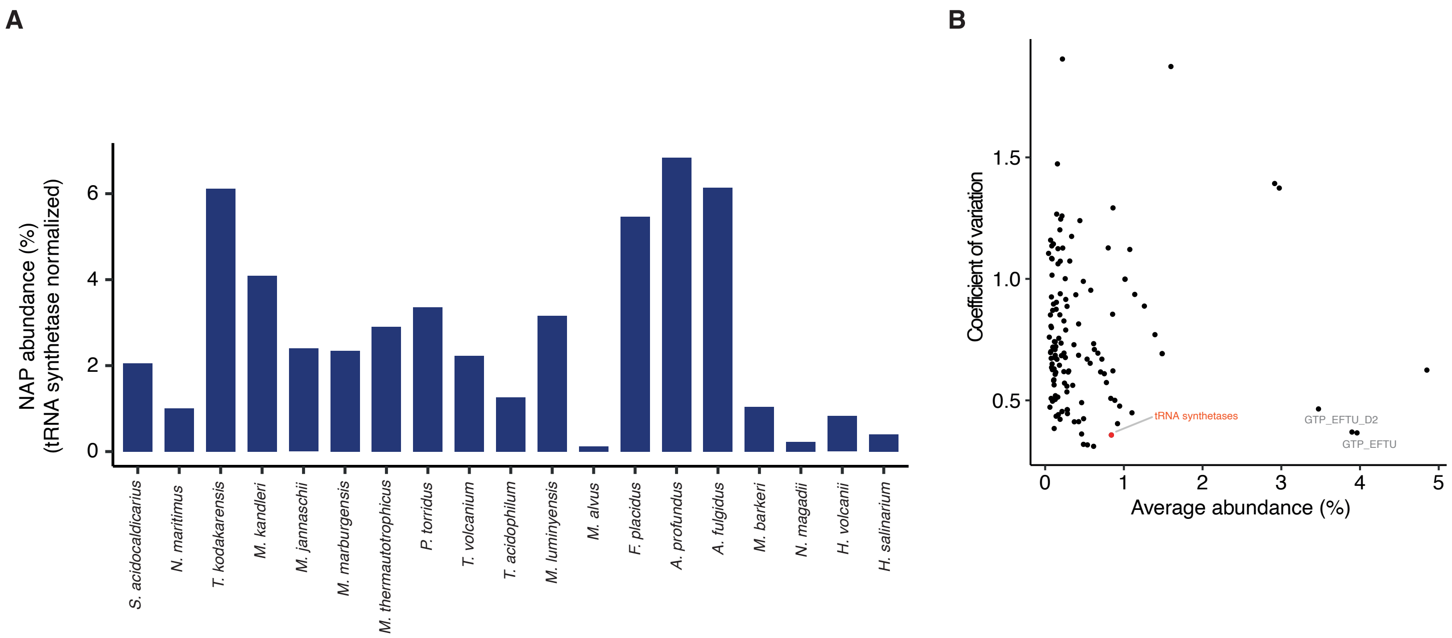

Figure S11. (**A**) Relative NAP abundance normalized to the abundance of tRNA synthetases in the same proteome. (**B**) Compared to other Pfam domains, proteins classified as tRNA synthetases are both highly abundant and different organisms dedicate a similar fraction of their protein budget to their production (as evident from a low coefficient of variation across species), making them a suitable choice for normalization. The idea of normalization here is to consider the investment in NAPs relative to some baseline investment in basic cellular processes (such as charging amino acids). The results indicate that large quantitative variability in NAP abundance is also evident when judged against this baseline rather than as a fractional allocation across the entire proteome.

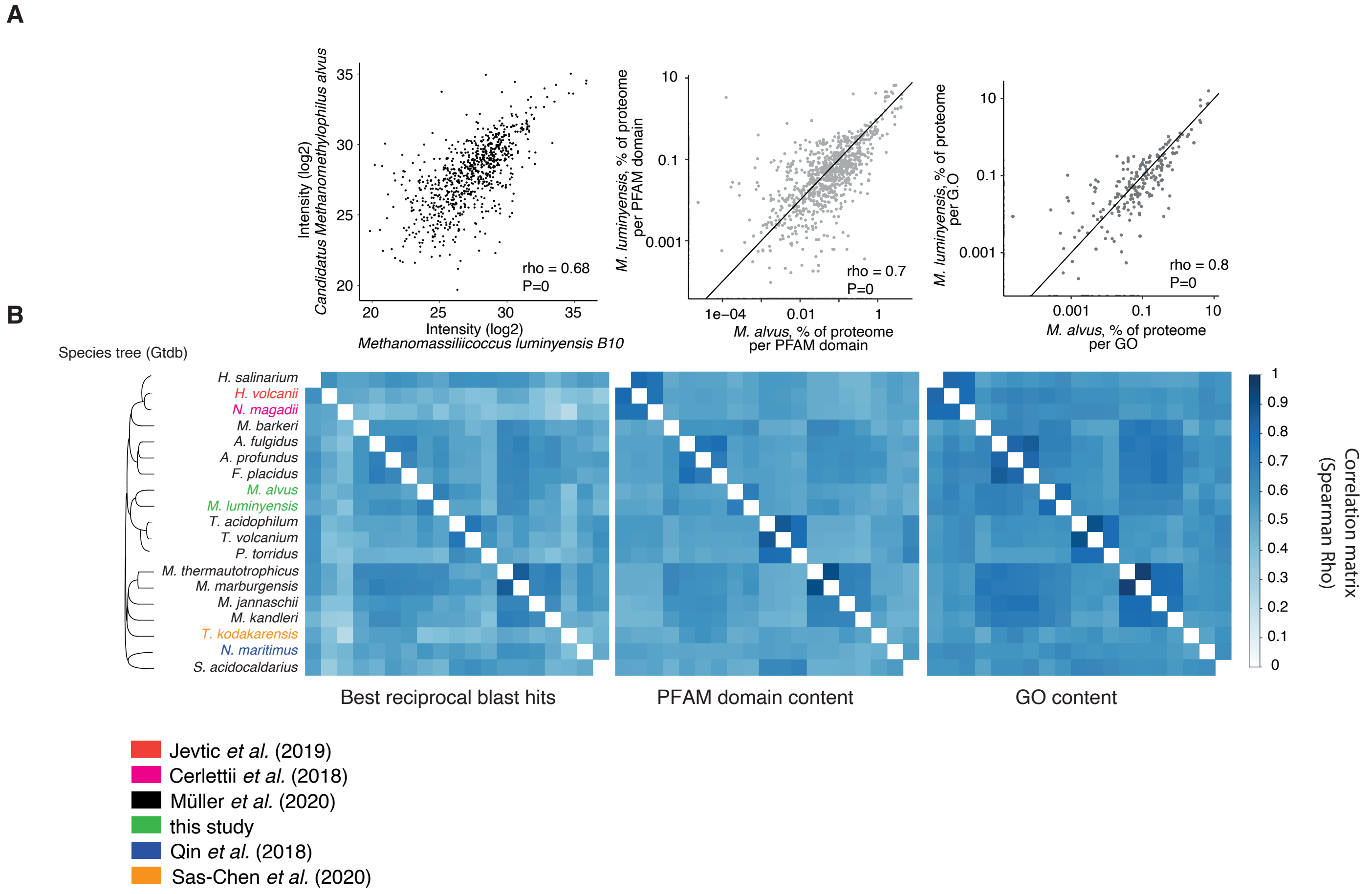

Figure S12. Comparing relative protein abundances across proteomes. (**A**) Correlation (Spearman's rho) of relative protein abundances in *Methanomassiliicoccus luminyensis* versus *Methanomethylophilus alvus* when comparing reciprocal best BLAST hits (left panel, n=775 proteins) or proteins aggregated by Pfam domain content (middle panel, n=3142 domains) or by gene ontology class (right panel, n=590 GO classes). (**B**) Visualization of pairwise correlation coefficients for the same comparisons across all 19 proteomes. The species tree is taken from GTDB, with *P. torridus* and *H. salinarum* added manually.

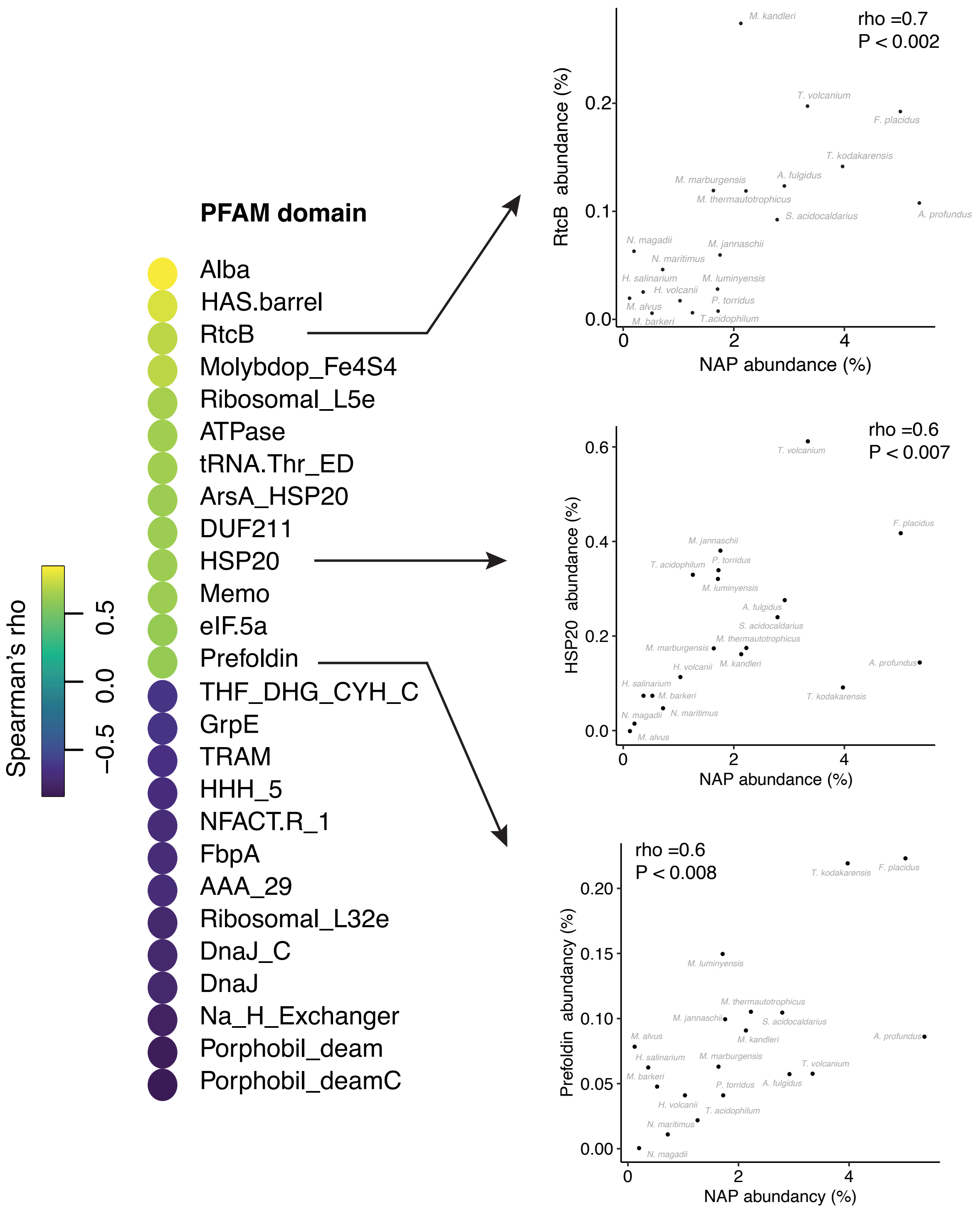

Figure S13. Top (bottom) 13 Pfam domains whose relative abundance is most positively (negatively) correlated (Spearman's rho) with relative NAP abundance (aggregated across known and candidate NAPs) across the sample of 19 archaea shown in Fig 2B. Examples of individual correlations are shown on the right (n=19 archaea in which domains were detected and abundance quantified).

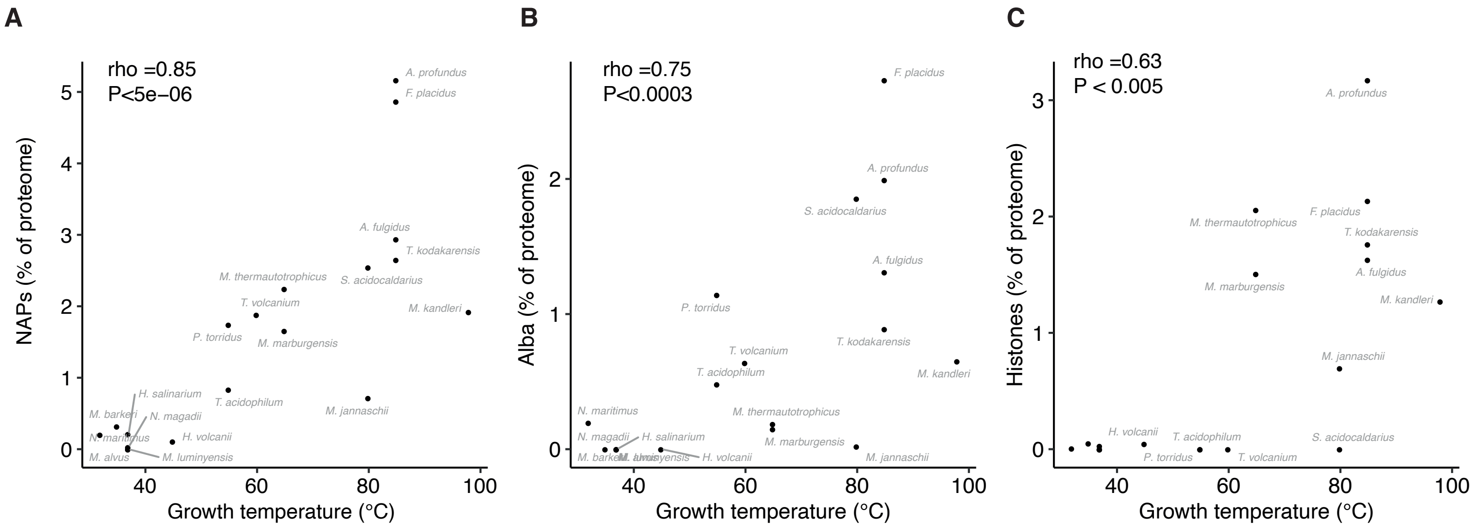

Figure S14. Relative abundance of individual NAPs as a function of optimal growth temperature. Strong correlations (Spearman's rho) are evident not only for (**A**) NAPs considered as an aggregate class (candidates excluded) but also for (**B**) Alba and (**C**) histones considered individually (n=19 archaea in which domains were detected and abundance quantified). Note that Alba and histones are the only NAPs that are sufficiently widespread across our sample of archaea to allow meaningful correlations to be computed.

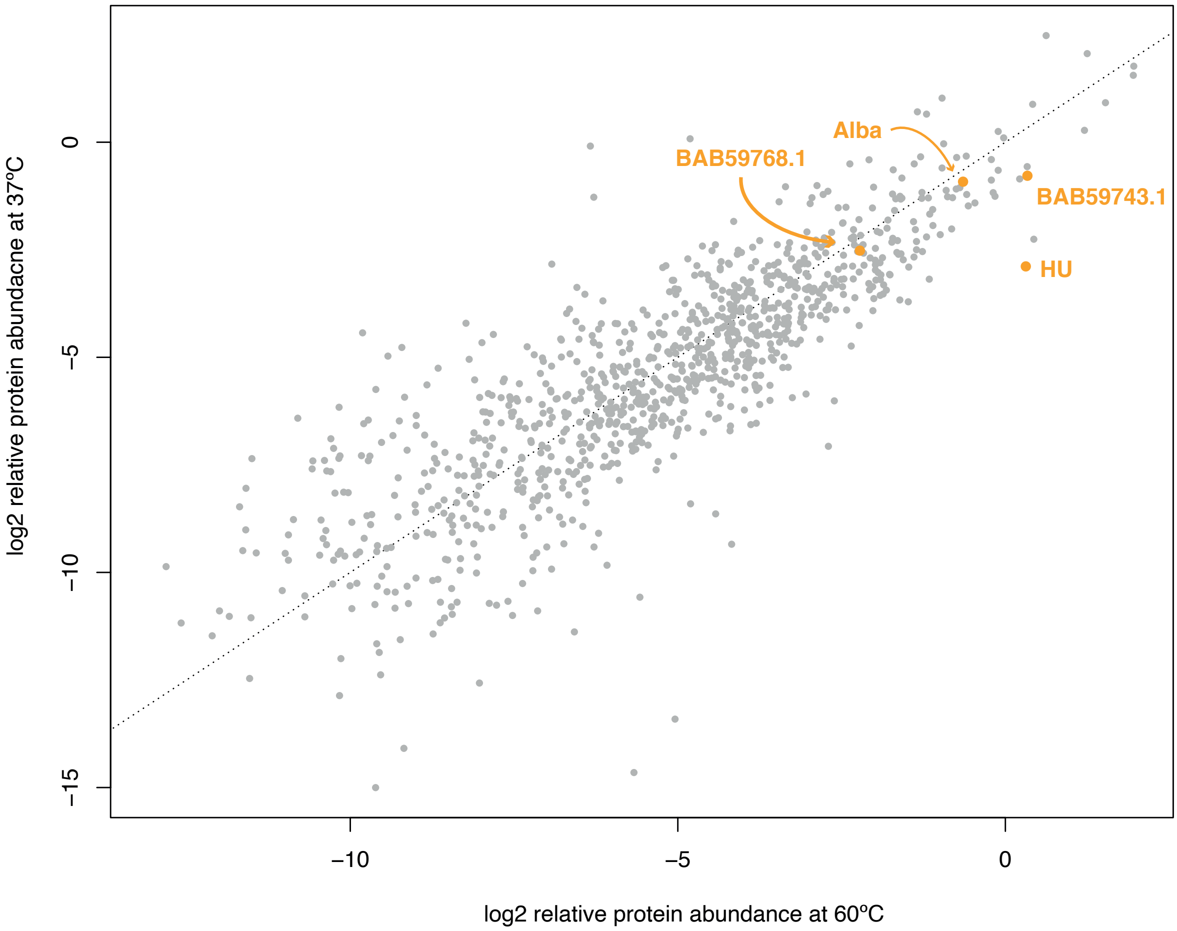

Figure S15. Protein abundances (whole cell extract) for *T. volcanium* grown at 60ºC and 37ºC.

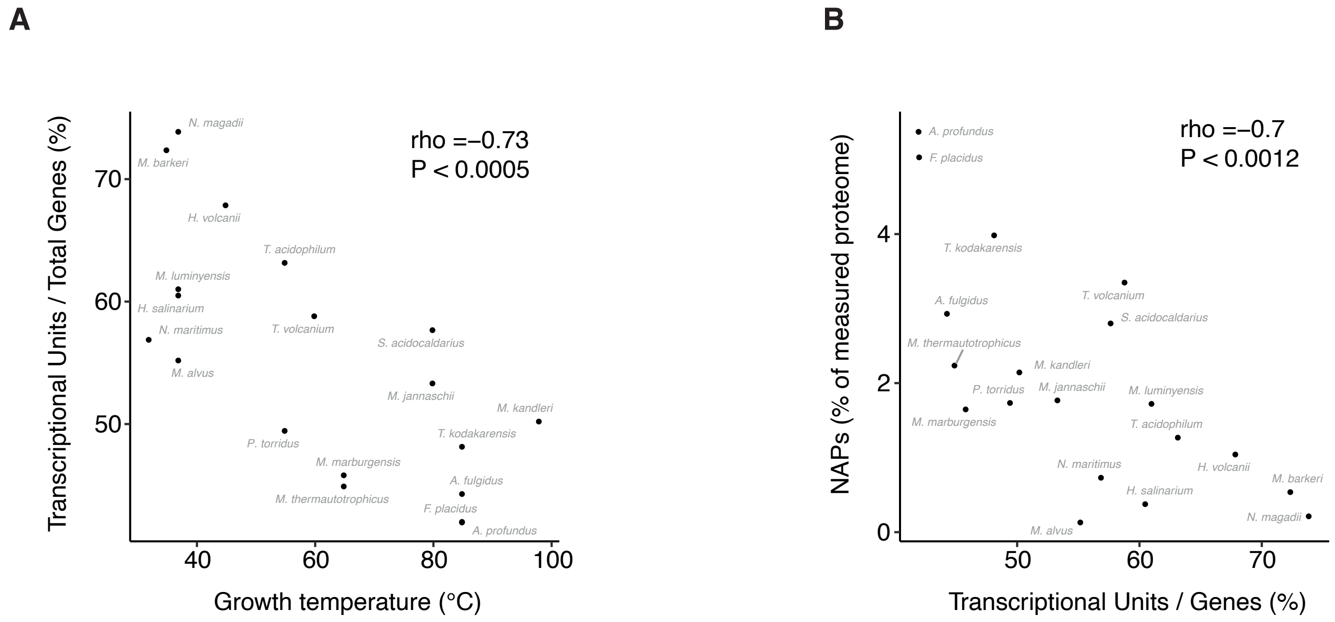

Figure S16. The relationship between growth temperature and genome compactness. (**A**) The genomes of organisms growing at higher temperatures tend to be organized in such a manner that transcript units harbour more genes on average. There are therefore fewer promoters per gene in archaea that grow at higher temperature. (**B**) As predicted from the respective relationships with optimal growth temperature, genomes where genes are contained in more independent transcriptional units have a lower investment in NAPs (Spearman's rho; n=19 archaea in which domains were detected and abundance quantified).
