# Supplementary material for: Growth temperature and chromatinization in archaea
Source: Nat Microbiol. 2022 Oct 20;7(11):1932–42. doi: 10.1038/s41564-022-01245-2 (PMC7613761; doi:10.1038/s41564-022-01245-2)
Supplement: Supplementary file 2 — Reporting Summary [file 41564_2022_1245_MOESM2_ESM.pdf]

nature portfolio

Corresponding author(s): Tobias Warnecke

Last updated by author(s): Aug 8, 2021

Reporting Summary

Nature Portfolio wishes to improve the reproducibility of the work that we publish. This form provides structure for consistency and transparency in reporting. For further information on Nature Portfolio policies, see our Editorial Policies and the Editorial Policy Checklist.

Statistics

For all statistical analyses, confirm that the following items are present in the figure legend, table legend, main text, or Methods section.

n/a	Confirmed	
☒	☐	The exact sample size (*n*) for each experimental group/condition, given as a discrete number and unit of measurement
☒	☐	A statement on whether measurements were taken from distinct samples or whether the same sample was measured repeatedly
☐	☒	The statistical test(s) used AND whether they are one- or two-sided<br>*Only common tests should be described solely by name; describe more complex techniques in the Methods section.*
☐	☒	A description of all covariates tested
☐	☒	A description of any assumptions or corrections, such as tests of normality and adjustment for multiple comparisons
☒	☐	A full description of the statistical parameters including central tendency (e.g. means) or other basic estimates (e.g. regression coefficient) AND variation (e.g. standard deviation) or associated estimates of uncertainty (e.g. confidence intervals)
☐	☒	For null hypothesis testing, the test statistic (e.g. *F*, *t*, *r*) with confidence intervals, effect sizes, degrees of freedom and *P* value noted<br>*Give P values as exact values whenever suitable.*
☒	☐	For Bayesian analysis, information on the choice of priors and Markov chain Monte Carlo settings
☒	☐	For hierarchical and complex designs, identification of the appropriate level for tests and full reporting of outcomes
☐	☒	Estimates of effect sizes (e.g. Cohen's *d*, Pearson's *r*), indicating how they were calculated

Our web collection on statistics for biologists contains articles on many of the points above.

Software and code

Policy information about availability of computer code

Data collection

Collection and analysis pipelines can be found at https://github.com/hocherantoine/NAPsQuant

Data analysis

Operons were predicted using https://biocomputo.ibt.unam.mx/operon_mapper/ with default settings (no version number provided by the authors of the software).
DNA binding was predicted using https://webs.iiitd.edu.in/raghava/dnabinder/ using the SVM model trained on a realistic dataset
Phylogenetic trees were visualized using https://itol.embl.de/
Archaeal trees were obtained from https://gtdb.ecogenomic.org
The Uniprot Retrieve/ID mapping tool was used for mapping IDs across proteomic/genomic data
Homolog detection was carried out using hmmsearch version 3.1b2 (option -ga) and jackhmmer (version 3.1b2)
Protein homologs were aligned using MAFFT (option -linsi) and phylogenetic trees built using RAXML-NG (model LG+R6)
To control for phylogenetic non-independence, phylogenetic linear regression were carried out using the R package phylolm, Model "BM" with 10000 bootstraps or 'OUrandomroot'. Variables were log transformed before regression.
Mass spectra were analyzed using the MaxQuant software platform (v1.6.10.43).

For manuscripts utilizing custom algorithms or software that are central to the research but not yet described in published literature, software must be made available to editors and reviewers. We strongly encourage code deposition in a community repository (e.g. GitHub). See the Nature Portfolio guidelines for submitting code & software for further information.

Data

Policy information about availability of data

All manuscripts must include a data availability statement. This statement should provide the following information, where applicable:

- Accession codes, unique identifiers, or web links for publicly available datasets
- A description of any restrictions on data availability
- For clinical datasets or third party data, please ensure that the statement adheres to our policy

All data generated or analyzed in this manuscript is publicly available as follows:
Optimal growth temperatures were obtained from bacdive-DSMZ (https://bacdive.dsmz.de/).
Genomes and predicted proteomes were obtained from https://www.ncbi.nlm.nih.gov/assembly. HMM models were downloaded from Pfam and TIGRFAMs 15.0. Gene ontologies were obtained from http://current.geneontology.org/ontology/external2go/pfam2go. Archaeal trees were obtained from https://gtdb.ecogenomic.org. Mass spectrometry data generated as part of this study have been deposited in the PRIDE repository with accession code PXD034568 (https://www.ebi.ac.uk/pride/archive/projects/PXD034568/).
Previously published data that were re-analyzed here and support the findings of this study are available as detailed in the original publications.
Correspondence or requests for materials should be address to AH (a.hocher@lms.mrc.ac.uk) or TW (tobias.warnecke@lms.mrc.ac.uk).

Field-specific reporting

Please select the one below that is the best fit for your research. If you are not sure, read the appropriate sections before making your selection.

☒ Life sciences ☐ Behavioural & social sciences ☐ Ecological, evolutionary & environmental sciences

For a reference copy of the document with all sections, see nature.com/documents/nr-reporting-summary-flat.pdf

Life sciences study design

All studies must disclose on these points even when the disclosure is negative.

Sample size	NA
Data exclusions	In collating publicly available archaeal proteomes, we only included proteomes that were derived a) from whole cell extract, b) without size selection and c) comprised more than 500 identified proteins.
Replication	Mass spectrometry experiments were carried out with two biological replicates for each condition (each with two technical replicates), except for *M. luminyensis* nucleoid enrichments (triplicates). Replicates for a given condition are highly correlated ($r^2>0.85$).
Randomization	NA
Blinding	NA

Reporting for specific materials, systems and methods

We require information from authors about some types of materials, experimental systems and methods used in many studies. Here, indicate whether each material, system or method listed is relevant to your study. If you are not sure if a list item applies to your research, read the appropriate section before selecting a response.

Materials & experimental systems

n/a	Involved in the study	
☒	☐	Antibodies
☒	☐	Eukaryotic cell lines
☒	☐	Palaeontology and archaeology
☒	☐	Animals and other organisms
☒	☐	Human research participants
☒	☐	Clinical data
☒	☐	Dual use research of concern

Methods

n/a	Involved in the study	
☒	☐	ChIP-seq
☒	☐	Flow cytometry
☒	☐	MRI-based neuroimaging
